# Ensemble modeling of SARS-CoV-2 immune dynamics in immunologically naïve rhesus macaques predicts that potent, early innate immune responses drive viral elimination

**DOI:** 10.3389/fimmu.2024.1426016

**Published:** 2024-11-07

**Authors:** Catherine Byrne, Joshua T. Schiffer

**Affiliations:** Vaccine and Infectious Disease Division, Fred Hutchinson Cancer Center, Seattle, WA, United States

**Keywords:** SARS-CoV-2, mathematical modeling, ensemble model, systems immunology, innate immunity, rhesus macaques, within-host infection dynamics

## Abstract

**Introduction:**

An unprecedented breadth of longitudinal viral and multi-scale immunological data has been gathered during SARS-CoV-2 infection. However, due to the high complexity, non-linearity, multi-dimensionality, mixed anatomic sampling, and possible autocorrelation of available immune data, it is challenging to identify the components of the innate and adaptive immune response that drive viral elimination. Novel mathematical models and analytical approaches are required to synthesize contemporaneously gathered cytokine, transcriptomic, flow cytometry, antibody response, and viral load data into a coherent story of viral control, and ultimately to discriminate drivers of mild versus severe infection.

**Methods:**

We investigated a dataset describing innate, SARS-CoV-2 specific T cell, and antibody responses in the lung during early and late stages of infection in immunologically naïve rhesus macaques. We used multi-model inference and ensemble modeling approaches from ecology and weather forecasting to compare and combine various competing models.

**Results and discussion:**

Model outputs suggest that the innate immune response plays a crucial role in controlling early infection, while SARS-CoV-2 specific CD4+ T cells correspond to later viral elimination, and anti-spike IgG antibodies do not impact viral dynamics. Among the numerous genes potentially contributing to the innate response, we identified IFI27 as most closely linked to viral load decline. A 90% knockdown of the innate response from our validated model resulted in a ~10-fold increase in peak viral load during infection. Our approach provides a novel methodological framework for future analyses of similar complex, non-linear multi-component immunologic data sets.

## 1 Introduction

The COVID-19 pandemic, caused by the novel coronavirus SARS-CoV-2, spurred an extraordinary global effort to comprehensively understand the pathophysiology of this infection. Longitudinal viral and multi-scale immunological datasets were amassed at an unparalleled scale (1–15), offering a unique opportunity to identify the intricacies of immune defense mechanisms during SARS-CoV-2 infection.

Animal models have proven especially useful for studying SARS-CoV-2 viral and immune kinetics (2, 3, 16–23). In contrast to the limitations faced in human studies, where sampling is often confined to saliva or nasal specimens collected after symptom onset, animal model studies enable sampling from the lung and other various tissue sites throughout the course of infection, including critical early pre-symptomatic time points. In addition, crucial variables such as time of infection, size of viral inoculum, viral variant, vaccination history, prior infection, and frequency of sampling are all experimentally controlled (24). Further, unlike in human studies where viral load is often the sole measure that is sampled longitudinally, animal models permit comprehensive assessments of changes in innate and adaptive immune responses over time (2, 3, 10, 22, 25, 26). SARS-CoV-2 infection in rhesus macaques presents relatively similarly to non-severe infection in humans, with comparable symptoms and duration of infection (10). As such, rhesus macaque data has proven particularly useful to better understand viral infection dynamics following vaccination, treatment, or reinfection with SARS-CoV-2 (2, 3, 10, 22, 23, 27, 28).

Despite the substantial volume of available data characterizing the SARS-CoV-2 immune response in rhesus macaques, there are no standardized methods to discern the relative importance and timing of mechanisms driving viral clearance. Immune responses demonstrate substantial redundancy (29). Moreover, separating and characterizing the dozens of gene products, antibodies, and immune cell populations which may be essential to infection clearance, presents computational challenges. Mathematical models of within-host infection dynamics fit to data offer a methodical way to test competing hypotheses of how a spreading infection and intensifying immune response may interact (30–33). Although numerous mathematical models of the within-host dynamics of SARS-CoV-2 infection have been developed to recapitulate viral load (11, 13, 33–38), only a few have concurrently integrated viral and immune data, and these have typically focused on a single arm of the immune system (35, 39–41). To our knowledge, no model has been fit concurrently to detailed longitudinal innate, cell-mediated, and humoral response data, with these responses variously coupled to SARS-CoV-2 elimination (42–44), and no model has focused specifically on lung immune responses.

When testing different mathematical models, multiple models may adequately fit observed, complex non-linear data such that several competing hypotheses to explain the data remain viable (45–47). This issue is compounded by the fact that even in carefully planned experiments, the sampling frequency may be too low during critical intervals to discriminate models with slightly differing assumptions. Consequently, testing, comparing, and amalgamating the outcomes of multiple potential models provides a more comprehensive and thorough result, allowing the weighting of multiple hypotheses and projecting necessary uncertainty into subsequent model predictions (48–50).

In the fields of climatology, ecology, and epidemiological modeling (51–57), there are methods for testing many different models and synthesizing predictions into ensemble models. This approach has yet to be adopted in within-host models of infectious disease but will be necessary to account for the rapid emergence of multi-component immune data. Whereas traditional viral dynamic models were fit to viral load alone, future models will be tasked to recapitulate viral load, gene signature data (11), T cell subset, B cell, innate immune cell, cytokine (58), and antibody levels (35) over time. Additional challenges will be weighing the strength of each data type for fitting and assessing for different degrees of misclassification across multiple assays. Akin to weather forecasts which attempt to predict precipitation, wind, and temperature, such detailed models can be utilized to predict viral and immune kinetics in individuals with different immune starting conditions due to prior vaccination or infection.

Here, using detailed rhesus macaque infection data, we develop and test 160 different mathematical models describing the virologic and immunologic dynamics of SARS-CoV-2 infection in the lung. Using the concepts of multi-model inference (50), we predict the importance, timing, and contribution of each tested immune control mechanism in clearing infected cells and free virus. Further, we develop ensemble model predictions that combine top model results and examine how these predictions diverge from those made by individual models. Together, our results provide further insight into the immune mechanisms of SARS-CoV-2 infection clearance, provide new methods for the analysis of similar data sets, but also highlight limitations of current methods to predict infection outcomes.

## 2 Results

### 2.1 Analysis of bronchoalveolar lavage fluid reveals dynamic immune and viral kinetics within the lung during SARS-CoV-2 infection

**Figure 1:**
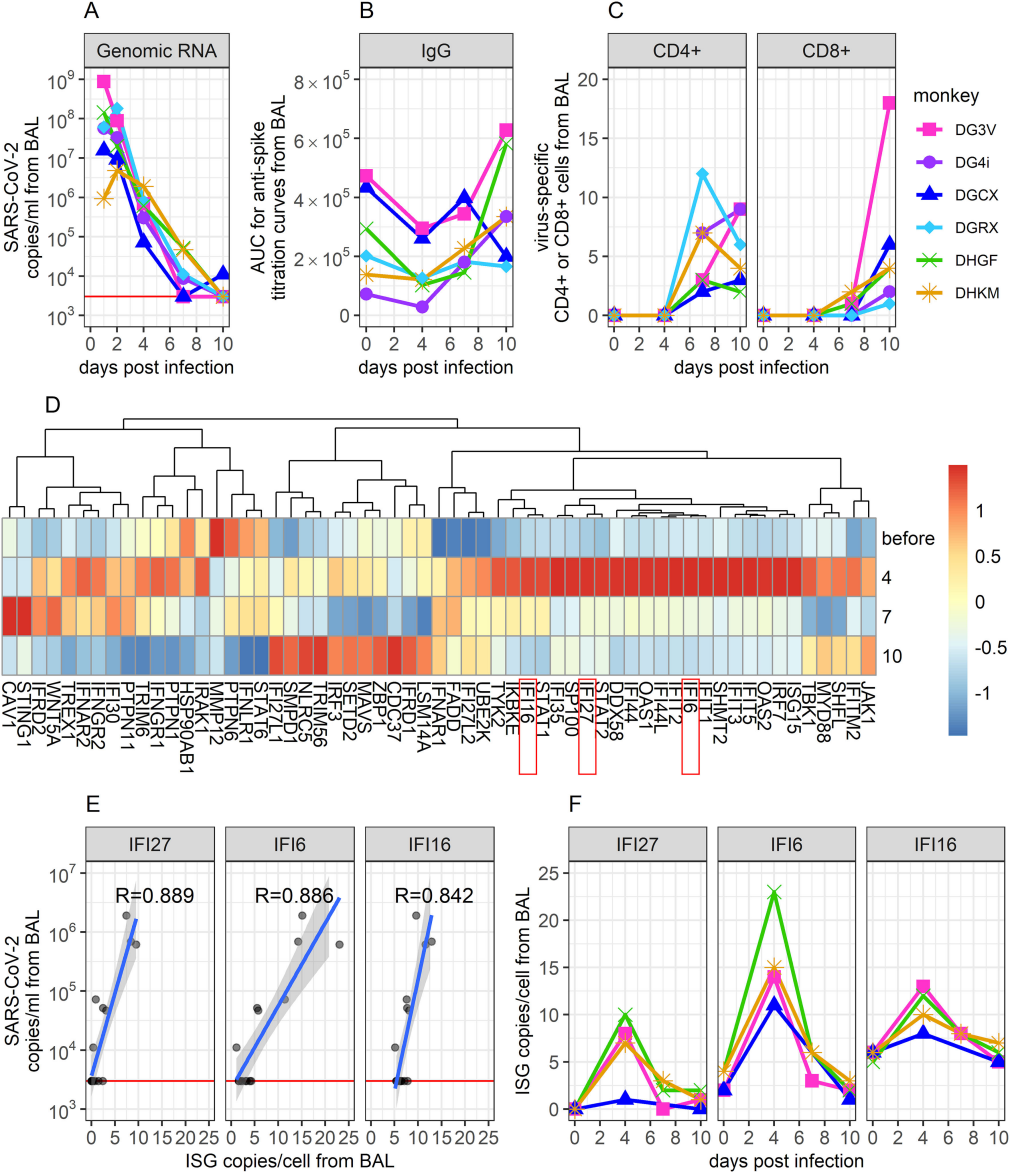
Description of viral and immunological data collected from BAL during SARS-CoV-2 infection in immunologically naïve Rhesus Macaques. **(A)** SARS-CoV-2 genomic (g)RNA copies/ml of BALF were measured using qPCR. The red horizontal line shows the threshold of detection (3000 copies/ml). Data points on days 1 and 2 are extrapolated from corresponding viral loads measured in the throat and the nose on these days (see Methods). **(B)** Area under the curve (AUC) from ELISA titration curves of SARS-CoV-2 anti-spike proteins in collected BALF. **(C)** Percentage of virus-specific CD4+ and CD8+ T cells in BALF, measured via flow cytometry. CD4+ and CD8+ cells were considered virus-specific if they stain positively for IFN γ or TNF following exposure to a megapool of SARS-CoV-2 antigens. **(D)** Normalized average expression values of IFN genes and ISGs, measured via scRNAseq. **(E)** Correlation between ISG expression and same-day gRNA levels in BALF. The three genes with the most significant correlation are shown (correlation coefficient indicated). These genes also showed the greatest change over time, according to feature selection. **(F)** Time-series data of these top three genes. Data comes from (2).

To obtain a representation of infection kinetics within the lung, Nelson et al. performed bronchoalveolar lavage (BAL) on SARS-CoV-2-infected rhesus macaques and analyzed BAL fluid (BALF) for viral genomic RNA concentrations with qPCR, anti-spike IgG titers with ELISA, SARS-CoV-2-specific CD4+ and CD8+ concentrations with cell staining and flow cytometry, and the expression of interferon (IFN) and IFN-stimulating genes (ISGs) with scRNAseq (2). We selected this dataset for modeling based on the longitudinal, multi-model measurements of immune response from the most relevant site of infection, the lung. Such data is not available from human studies. The data was notable for a decline in SARS-CoV-2 RNA starting on day 1 (**Figure 1A**), relatively stable anti-spike IgG levels with a slight increase in some animals (**Figure 1B**), late increases in virus-specific CD4+ and CD8+ T cells (**Figure 1C**), and early increases and abrupt decreases in multiple innate gene signatures (**Figure 1D**).

While wanting to describe as much of these data as possible within our mathematical model, it was unfeasible to mechanistically model the dynamics of all genes examined through scRNAseq without making the model unworkably complex. To identify genes of interest, we performed feature selection (59) on IFN and ISG time series data and examined how gene expression correlated with same-day viral loads. Results indicated that the IFI27, IFI6, and IFI16 genes showed the strongest correlations with viral load (**Figure 1E**) and the greatest variability over time (**Figure 1F**). In addition, these genes were selected based on mechanistic importance and high dynamics during human SARS-CoV-2 infection (60–67). Thus, in addition to viral genomic RNA concentrations, anti-spike IgG titers, and virus-specific CD4+ and CD8+ concentrations, we included the dynamics of these three genes in our mathematical model of infection. As ISGs are known to mediate the innate immune response (8, 68), these three genes were assumed to capture the dynamics of the innate immune response and describe its impact on viral dynamics within our model.

### 2.2 Competing mathematical models of immune containment SARS-CoV-2 infection

**Figure 2:**
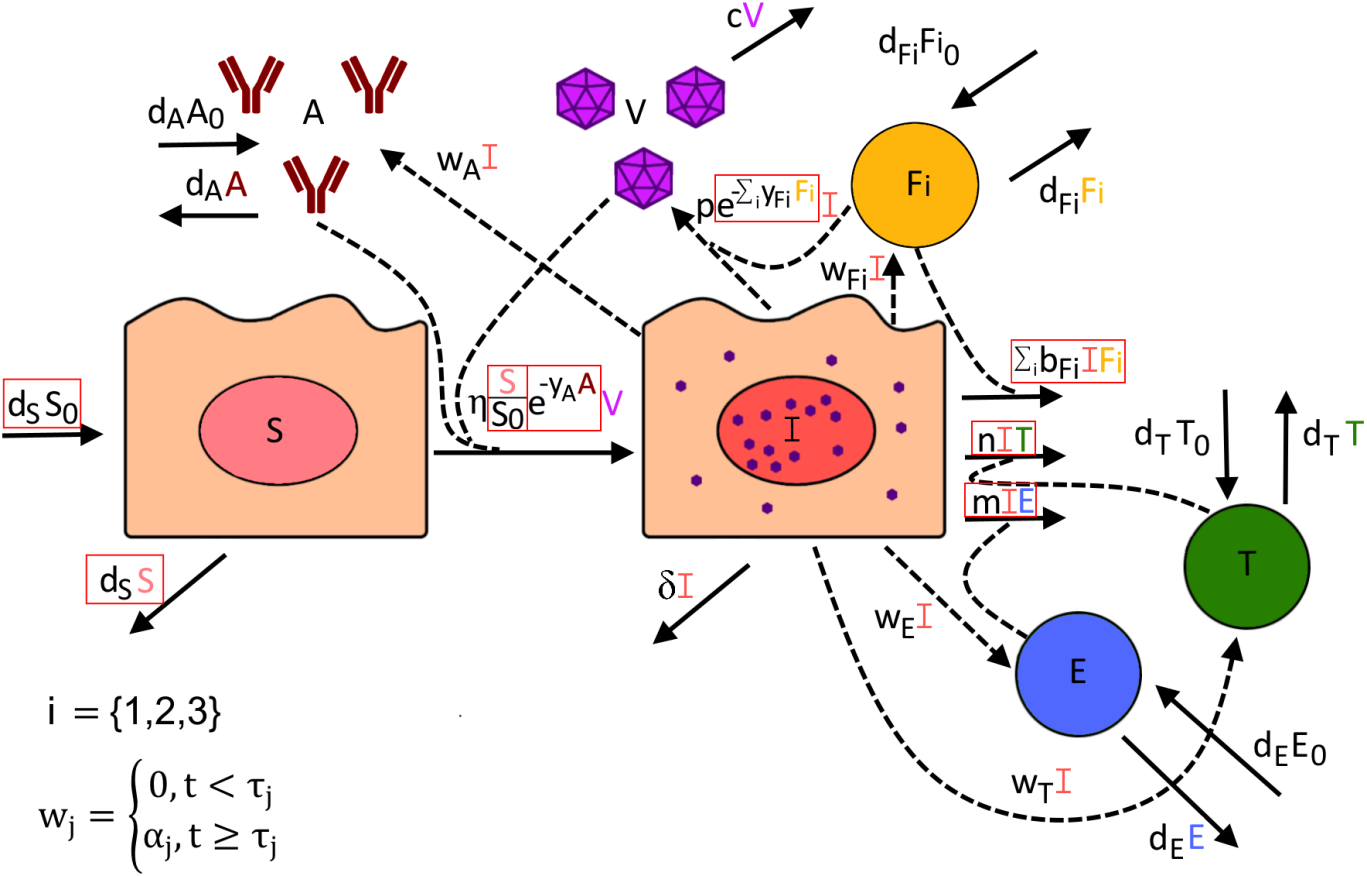
Visual description of all potential terms included within our mathematical model of SARS-CoV-2 infection. Red boxes indicate which model terms were alternatively included or excluded to determine how well each version of this model fits the data. The rate at which susceptible cells (S) become infected (I) is dependent on the number of susceptible cells, the amount of virus (V) present, and the presence of anti-spike IgG (A), which may dampen infection rates through neutralization of virus. Infected cells can potentially be cleared by interacting with virus-specific CD8+ T cells I, virus-specific CD4+ T cells (T), or the innate immune response ( Fi ). The rate of viral production is dependent on the number of infected cells but can be dampened by the innate immune response inducing an antiviral state in infected cells. The rate of proliferation for anti-spike IgG antibody, virus-specific CD8+ T cells, virus-specific CD4+ T cells, and innate immune cells is proportional to the number of infected cells but is not turned on until time $\tau_{j}$ , where j is specific to the type of immune response. For the innate immune response ( Fi ), we test its dynamics being represented by three possible ISGs: IFN27 (i=1), IFI6 (i=2), and IFI16 (i=3).


Many models of within-host dynamics during SARS-CoV-2 infections exist (11, 13, 33–37), but few incorporate detailed data on how different arms of the immune response might impact infection (35, 39–41). To test the role of both the innate and adaptive immune response on SARS-CoV-2 elimination, we developed the following model (**Figure 2**) describing how the kinetics of infection within the lung change over time, t .

$$\frac{dS}{dt}=d_{S}S_{0}-\eta \frac{S}{S_{0}}e^{-y_{A}A}V\;-\;d_{S}\;S$$

$$\frac{dI}{dt}=\eta \frac{S}{S_{0}}e^{-y_{A}A}V-\sum_{i}b_{Fi}IF_{i}-nIT-mIE-\delta I$$

$$\frac{dV}{dt}=pe^{-\sum_{i}y_{Fi}F_{i}}I-cV$$

$$\frac{dFi}{dt}=d_{Fi}Fi_{0}+w_{Fi}I-d_{Fi}Fi$$

$$\frac{dT}{dt}=d_{T}T_{0}+w_{T}I-d_{T}T$$

$$\frac{dE}{dt}=d_{E}E_{0}+w_{E}I-d_{E}E$$

$$\frac{dA}{dt}=d_{A}A_{0}+w_{A}I-d_{A}A$$

where


i={1,2,3}

and


$$w_{j}=\;\{\begin{array}{c} 0,\;t<\tau_{j}\\ \alpha_{j},\;t\geq \tau_{j} \end{array}$$

In this model, susceptible cells ( S ) are produced at a constant rate $d_{S}S_{0}$ , where $S_{0}$ is the number of susceptible cells in the lung before infection ( t=0 ), and naturally die at a rate $d_{s}S$ . These cells become infected by SARS-CoV-2 virions ( V ) at rate $\eta \frac{S}{S_{0}}e^{-y_{A}A}V$ and move into the infected compartment ( I ). This rate incorporates the potential role of anti-spike IgG ( A) in dampening infection rates through virus neutralization, with the scaling factor $e^{-y_{A}A}$ taking on a value between 0 and 1. Thus, higher IgG antibody levels may lower infection rates according to their concentration.

Virus-specific CD4+ T cells ( T ), virus-specific CD8+ T cells ( E ), the innate immune response captured by IFI27, IFI6, and IFI16 expression ( F1, F2, F3 , respectively), and anti-spike IgG each are assumed to have constant rates of production and per-capita natural death rates that maintain an equilibrium in the absence of infection. Infection also stimulates the production of each compartment, with the rate of proliferation proportional to the number of infected cells. We, however, assume that infection-induced proliferation for each compartment remains zero until time $\tau_{j}$ at which point onwards it is equal to $\alpha_{j}I$ , where j is specific to the immune compartment.

Infected cells may be cleared by the innate immune response, virus-specific CD4+ T cells, or virus-specific CD8+ T cells, at rates $\sum_{i}b_{Fi}IFi$ , nIT , and mIE , respectively. Infected cells may also naturally die at a per-capita rate δ . Virus is produced by infected cells at a per-capita rate p which may be dampened by the innate immune response by a factor $e^{-\sum_{i}y_{Fi}F_{i}}$ . Virus is naturally cleared at a per-capita rate c .

To test different versions of the model, we alternated setting parameters $b_{F1},\;b_{F2},\;b_{F3},\;n,\;m,\;y_{F1},\;y_{F2},\;y_{F3}$ and $y_{A}$ to zero as well as including or excluding potential target cell limitation, where when excluded susceptible cells were not modeled and the infectivity term $\eta \frac{S}{S_{0}}e^{-y_{A}A}V$ was set to $\eta e^{-y_{A}A}V$ . As the impact of the separate ISGs is likely hard to distinguish, we only allowed one ISG at a time to have an impact on clearing infection and/or dampening viral production. With these combinations, a total of 160 versions of this model were developed and fit to available data (**Figure 1**), with appropriate transformations to make each set of data representative of counts within BALF (see **Supplementary Material** for further details).

### 2.3 Ensemble modeling to synthesize multiple well-fitting mathematical model versions for multi-model inference

Upon fitting each version of our mathematical model (described in Methods), we calculated Akaike Information Criterion (AIC) scores to determine how well each model performed. Here, the AIC score for model j is defined as


$$AIC_{j}=2k_{j}-2\text{ln}\left(L_{j}\right)$$

where $k_{j}$ is the number of model-estimated parameters in model j and $L_{j}$ is model j ’s maximized likelihood value.

**Figure 3:**
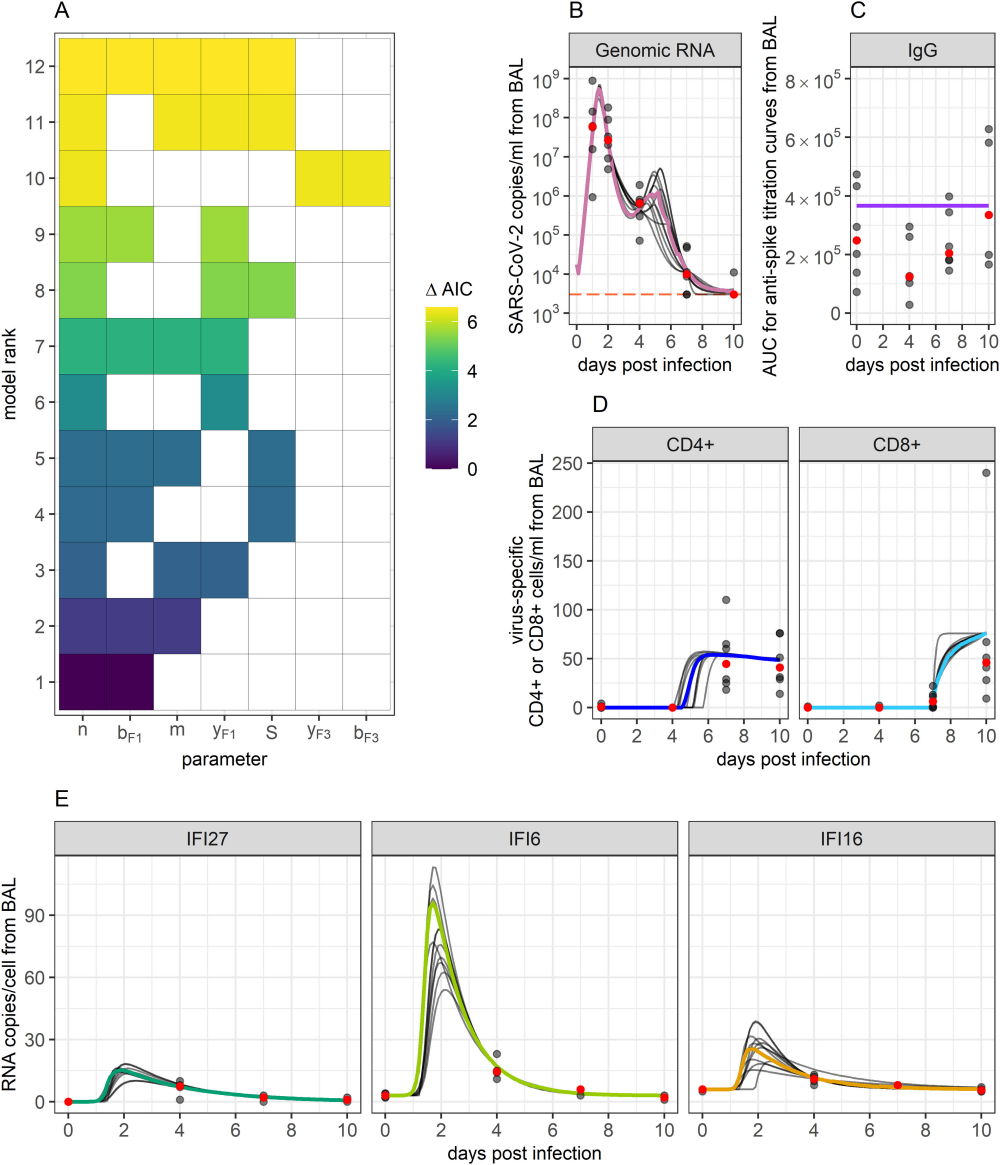
Ensemble model fits to SARS-CoV-2 viral and immune data. **(A)** Ranking of the top 12 models that best fit biological data and whose AIC scores add up to a summed weight of 0.95. The X-axis displays which of the tested parameters were included within a particular model, while the Y-axis ranks models based on their AIC scores. Term “S” represents the inclusion of target cell limitation. AIC scores are indicated by the color of the filled-in boxes. These top 12 models were used to create an ensemble model to capture the combined results. **(B–E)** Ensemble weighted median (colored lines) and the individual top 12 model fits (grey lines) to data. Grey dots indicate data points. Red dots indicate the median of the data points. Orange horizontal line indicates the threshold of detection for qPCR.

Based on AIC scores, each model was assigned a rank, with lower AIC scores corresponding to a better model. In examining the dynamics of all top-ranked models, we noted that they all predicted the size of the anti-spike IgG population to remain unchanged over the 10-day study (**Figure 3C**). This comes from the data being relatively flat with only a slight increase noted after viral elimination, as has been observed in infection of immunologically naïve humans (35). As such, the $e^{-y_{A}A}$ component within the model’s infection term ( $\eta \frac{S}{S_{0}}e^{-y_{A}A}V$ ) remained constant over time and model results including this term were indistinguishable from models where this constant was incorporated into the value of η . As such, we chose to exclude all models that did not set $y_{A}$ to zero from our analysis, reducing the total number of models to 80. The ranking of all 80 models appears in the **Supplementary Material** (**Supplementary Figure S2**).

We compared models through several quantifiers. We calculated AIC score differences ( $\text{Δ}AIC_{j}$ ), capturing the difference between the AIC score of model j and the minimum AIC score across all models, the evidence ratios ( $ER_{j}$ ), capturing how much more likely the model with the lowest AIC score is to model j , and AIC weights ( $w_{j}$ ), capturing the probability that model j best captured the data. Definitions of these terms are provided in the Methods.

Values for the top-ranked models are displayed in **Table 1**. The AIC weights of the top 12 ranked models (**Figure 3A** and **Table 1**) summed to 0.95, thus giving us the 95% confidence set that we used for further analysis. Among these 12 models, likelihood and AIC scores were relatively similar, with no single model being overwhelmingly the best. However, the models’ Akaike weights indicate large variability in how probable each was at best capturing the data. For example, while the top-ranked model has a probability of 0.30 for best-capturing data, the probability is 0.012 for the 10^th^-ranked model, making the first model 25 times more likely.

**Table 1:** 95% confidence set of best-ranked models describing SARS-CoV-2 infection in Rhesus Macaques.

Rank	Included Parameters/ Compartments	ln( $L_{j}$ )	$k_{j}$	$AIC_{j}$	$\Delta AIC_{j}$	$ER_{j}$	$w_{j}$	Summed Weight
1	$n,\;b_{F1}$	-741.273	23	1528.547	0.000	1.000	0.305	0.305
2	$n,\;b_{F1},\;m$	-740.845	24	1529.691	1.144	1.771	0.172	0.477
3	$n,\;m,\;y_{F1}$	-741.292	24	1530.584	2.038	2.770	0.110	0.587
4	$n,\;b_{F1},\;S$	-741.392	24	1530.784	2.237	3.061	0.100	0.686
5	$n,\;b_{F1},m,\;S$	-740.433	25	1530.865	2.318	3.187	0.096	0.782
6	$n,\;y_{F1}$	-742.860	23	1531.720	3.173	4.887	0.062	0.844
7	$n,\;b_{F1},m,y_{F1}$	-741.381	25	1532.763	4.216	8.231	0.037	0.882
8	$n,\;y_{F1},\;S$	-742.958	24	1533.916	5.369	14.648	0.021	0.902
9	$n,\;b_{F1},\;y_{F1}$	-743.086	24	1534.172	5.625	16.650	0.018	0.921
10	$n,\;y_{F3},b_{F3}$	-743.486	24	1534.972	6.425	24.847	0.012	0.933
11	$n,\;m,y_{F1},\;S$	-742.507	25	1535.014	6.467	25.370	0.012	0.945
12	$n,\;b_{F1},\;m,y_{F1},\;S$	-741.568	26	1535.136	6.589	26.964	0.011	0.956

Here, for each model j within the confidence set, the ln of the likelihood ( $L_{j}$ ), the number of free parameters ( $k_{j}$ ), the Akaike information criterion score ( $AIC_{j}$ ), the evidence ratio ( $ER_{j}$ ), the weight ( $w_{j}$ ) and the summed weight is displayed. The tested model parameters that were included in each model are listed. Models were ranked in order of their AIC scores.

To capture the combined predictions of our different models, we developed an ensemble model. While ensemble modelling can encompass a host of different ways of combining model predictions (51–57), here we define ours as the Akaike-weighted median behaviors of the 95% confidence set over time. As such, this model tracks the median and confidence intervals of all viral and immune variables exhibiting dynamics within the observation window. By choosing the 95% confidence set of models, we are 95% confident that one of the models we have included is the best at approximating the data (50). The ensemble model’s predictions of each model compartment’s dynamics over time are displayed in **Figures 3B–E**, along with projections of each of the top 12 models. Our ensemble model predicted there to be a period of very slight viral rebound occurring around days 4 and 5 post-infection. Viral loads were predicted to rebound from a local minimum of $3.3\times 10^{5}$ copies/ml of BALF at 3.6 days post-infection to a local maximum of $1.3\times 10^{6}$ copies/ml of BALF at 5.2 days post-infection, before continuing back downwards. This resurgence in virus corresponds with a decreasing innate response, while eventual re-control corresponds to rising T cell responses. The model also predicted a surge in innate responses peaking slightly after maximal viral load at comparable orders of magnitude for IFI27, IFI16 and IFI6 relative to baseline to those in humans (11, 69).

### 2.4 Importance and timing of the innate and adaptive immune response in clearing SARS-CoV-2 infection

Using Akaike weights from the models within the 95% confidence set, we calculated how important each tested model term was to obtaining optimal fit to viral and immune dynamic measures during SARS-CoV-2 infection (**Table 2**). To calculate importance, we summed the Akaike weights of the models in which each term appeared and normalized by the total accumulated weights of the 95% confidence set. We repeated this analysis assuming a lower cut off for late viral loads and detected no significant differences in model selection (not shown).

**Table 2:** The relative importance of tested parameters in capturing SARS-CoV-2 rhesus macaque infection dynamics.

rameters/Compartment	Description	Importance
$b_{F1}$ , $b_{F3},\;y_{F1},y_{F3}$	Impact of the innate response on infection	1
n	Infected cell clearance by CD4+ T cells	0.95
$b_{F1}$	Infected cell clearance by IFI27	0.74
m	Infected cell clearance by CD8s	0.44
$y_{F1}$	Hindrance of viral production by IFI27	0.27
S	Target cell limitation	0.24
$y_{F3}$	Hindrance of viral production by IFI6	0.012
$b_{F3}$	Infected cell clearance by IFI6	0.012
$b_{F2}$	Infected cell clearance by IFI16	0
$y_{F2}$	Hindrance of viral production by IFI16	0
$y_{A}$	Hindrance of infection by anti-spike IgG	0

Importance is determined by summing the Akaike weights of the 95% confidence set of best-ranked models for which a particular parameter appears.

Our results indicate that parameters describing the impact of the innate immune response on infection were essential to describe observed viral and immune data, appearing in every model within the 95% confidence set, and thus had an importance of 1. Of these innate immune parameters, infected cell clearance by IFI27 appeared most frequently, having an importance of 0.74. Infected cell clearance by virus-specific CD4+ T cells also ranked highly having an importance of 0.95, while infected cell clearance by virus-specific CD8+ T cells had an importance of 0.44. The inclusion of target cell limitation within the model had an importance of 0.24.

**Figure 4:**
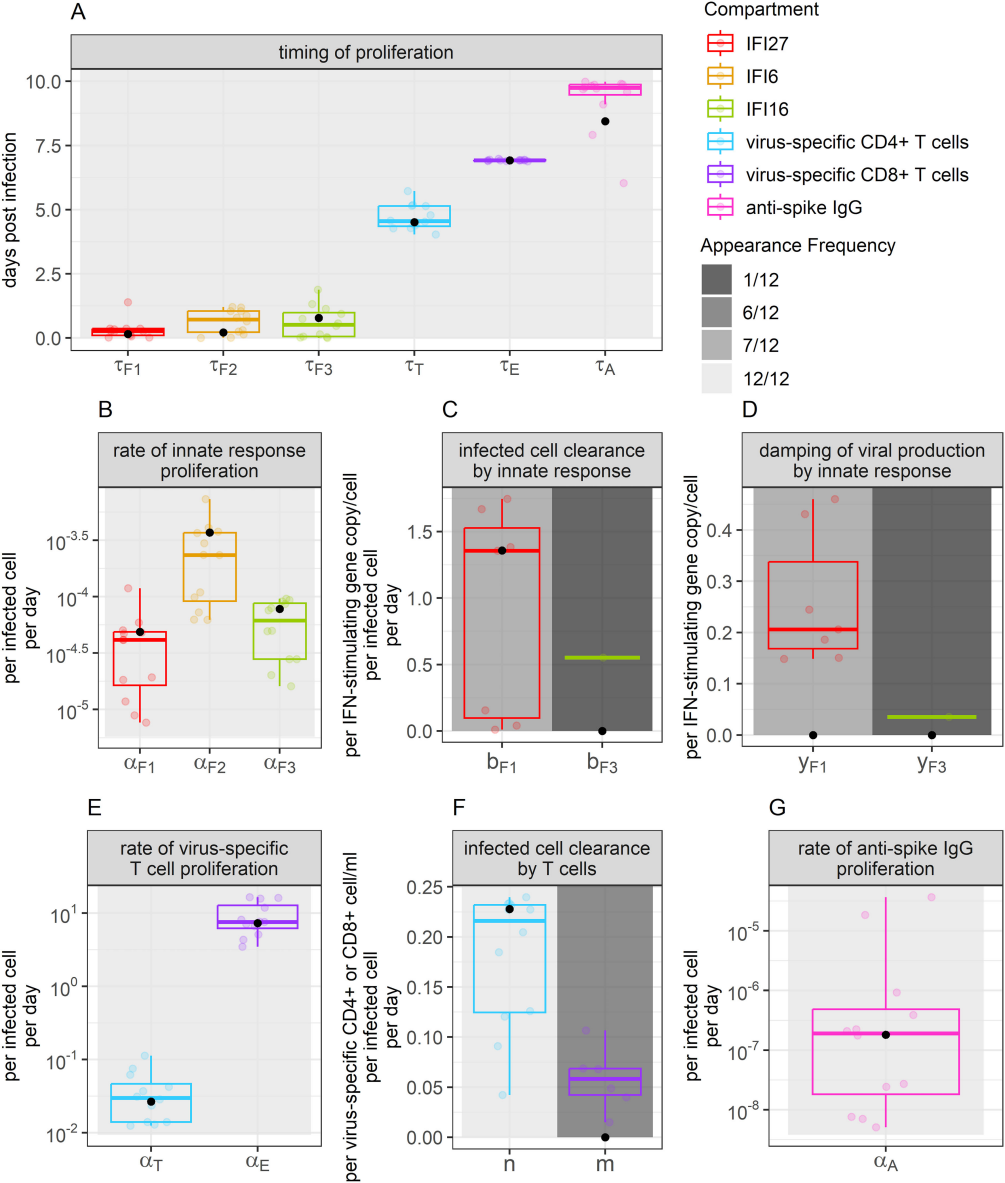
Parameter values appearing for the best-ranked models. Boxplot color indicates the immune compartment to which a parameter belongs. Background shade indicates how often a parameter appeared within the 12 best-ranked models. Colored dots show individual parameter values for the models that include each parameter (value not set to 0). Boxplots show the median and interquartile range (IQR), while whiskers indicate 1.5 times the IQR. Black dots indicate the weighted median of each parameter value, determined using model Akaike weights when accounting for all models in the 95% confidence set. Panel **(A)** displays values related to the timing of proliferation. Panel **(B)** displays values related to innate immune response proliferation. Panels **(C, D)** display values related to the clearance of infected cells and damping of viral production by the innate response, respectively. Panels **(E, F)** display values related to the proliferation of, and clearance of infection by T cells, respectively. Panel **(G)** displays values describing the rate of anti-spike IgG proliferation.

Parameter values from the 95% confidence set were relatively preserved across models (**Figure 4**). As our model predicts when the proliferation of each immune compartment begins, we developed a timeline of how infection is eliminated. Our model suggests that interferon expression, characterizing the innate immune response, expands early during infection. IFI27, IFI6, and IFI16 expansion were predicted to initiate a median of 0.27, 0.72 and 0.52 days post-infection, respectively. This was followed by increases in virus-specific CD4+ T cells beginning at 4.5 days post-infection. Virus-specific CD8+ T cell proliferation was predicted to begin 6.9 days post-infection. Anti-spike IgG antibodies were predicted to increase a median of 9.7 days post-infection; however, IgG expansion rates were very low with the AUC under titration curves expected to increase by a median of $1.9\times 10^{-7}$ per infected cell per day. By this time point, BALF viral loads in 5 out of the 6 monkeys were below the threshold of detection, providing further support that these antibodies had little role in controlling primary infection in this immunologically naïve cohort.

When comparing the models that include infection clearance by the different ISGs, we identified that the per-capita clearance rate of infected cells is a median of 2.5 times faster per IFI27 gene expressed than it is per IFI6 gene expressed ( $b_{F1}/b_{F3}$ ). Further, we found that the coefficient $y_{Fi}$ , allowing for dampening of viral production, is a median of 5.8 times larger per IFI27 gene expressed than it is per IFI6 gene expressed ( $y_{F1}/y_{F3}$ ). None of the models in the 95% confidence set included the role of infection clearance or dampening of viral production by IFI16. When performing a similar comparison for the models that included infection clearance by T cells, we found that the per-capita clearance rate of infected cells was a median of 3.7 times faster per virus-specific CD4+ T cell/ml than it was per virus-specific CD8+ T cell/ml in the BALF ( m/n) .

The values of the remaining parameters in our model appear in the **Supplementary Material** (**Supplementary Figure S3**), as does an analysis of the correlation between fitted parameter values (**Supplementary Figure S4**). Notably, some parameter values are positively and negatively correlated indicating a potential lack of complete mathematical identifiability. Alternatively, these correlations may have a mechanistic underpinning. For instance, the three cytokines often proliferate at correlated rates which could reflect an equivalent cellular source; cytokine proliferation often correlates with enhanced cell killing by cytokines as well as CD4+ T cells which may suggest coupled innate and acquired immune responses.

**Figure 5:**
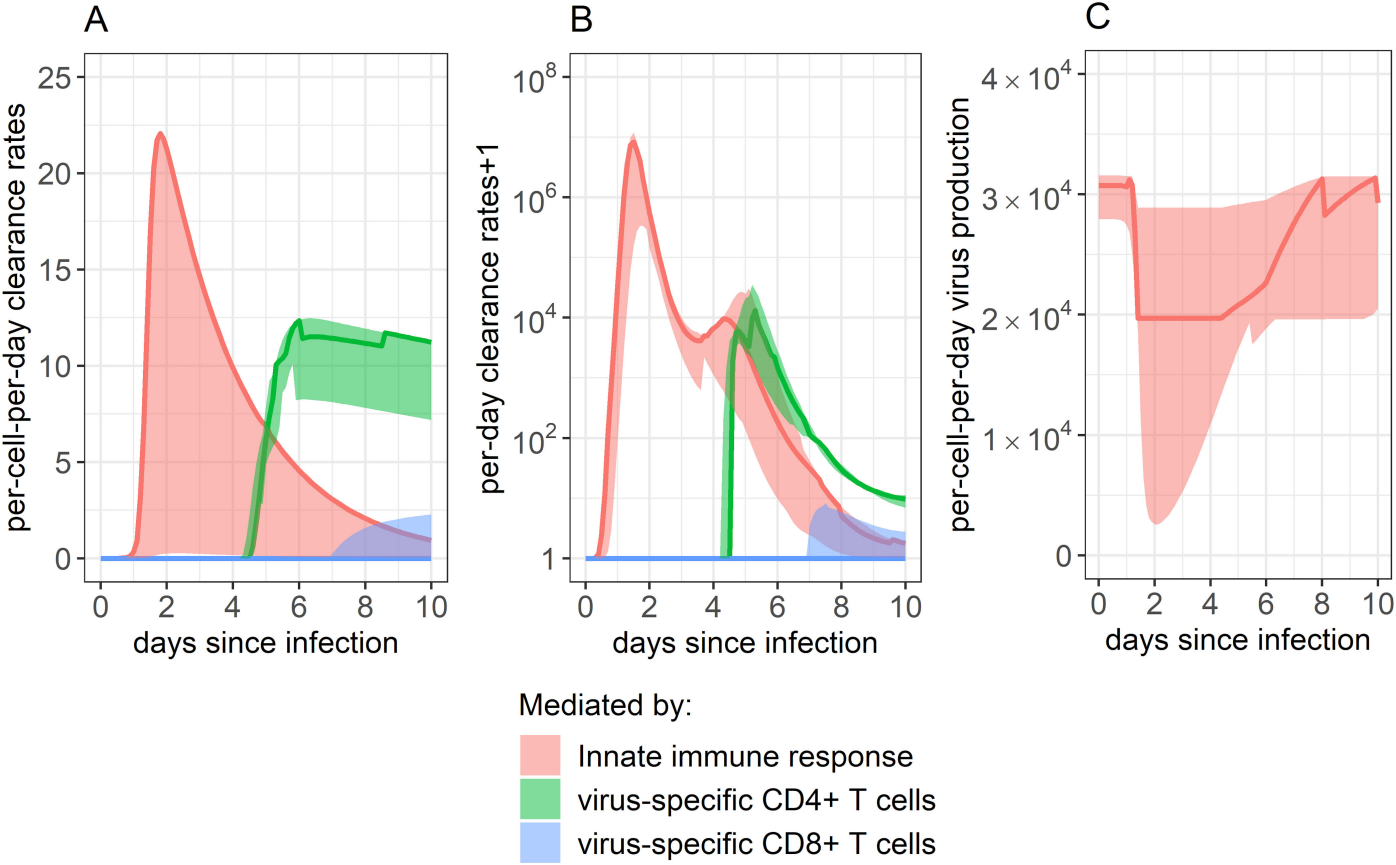
Impact of each immune component in eliminating SARS-CoV-2 infected cells. **(A, B)** Rate of infected cell clearance throughout infection, as mitigated by the innate immune response, CD4+ T cell response, and CD8+ T cell response. Panel A shows the per-infected cell-per-day clearance, while panel B shows the total per-day clearance rate. Note that panel B shows the “per-day clearance rate+1” so that values may be displayed on a log scale. **(C)** Innate immune system’s impact on dampening the rate of viral production by inducing an antiviral state in infected cells. The innate immune system’s impact was determined by combining the impact of all ISGs examined. As our best-fitting models did not include any impact of anti-spike IgG on infection control, their role is not displayed here. Solid lines show the weighted median, while ribbons show the weighted interquartile range, calculated using the Akaike weights from the 95% confidence set of best-ranked models.

We next used our 95% confidence set of models to examine how the rates of infected cell clearance (**Figures 5A, B**) and virus production (**Figure 5C**) fluctuated throughout the 10-day study period as a function of the different arms of the immune response. We identified that the rate of infected cell clearance is predicted to peak around day 2 post-infection. Clearance of infected cells was predicted to depend solely on the innate immune response for the first 4 days post-infection. Between days 4 and 5 post-infection, the dominating immune response switched, with virus-specific CD4+ T cells increasing and appearing most closely linked with infected cell lowered viral load. Around 7 days post-infection, some remaining clearance was predicted to be attributed to the virus-specific CD8+ response, though the role of virus-specific CD4+ T cells continued to predominate.

**Figure 5C** shows how the rate of virus production per-infected-cell-per-day changes over time as a function of the innate immune response. Each infected cell was predicted to produce the lowest amount of virus per day around day 2 post-infection when ISG expression was highest (**Figure 3E**), with rates at their lowest being a median of 64% of what they were before the innate immune response began to proliferate.

### 2.5 Projections of infection assuming weaker innate and T-cell responses

**Figure 6:**
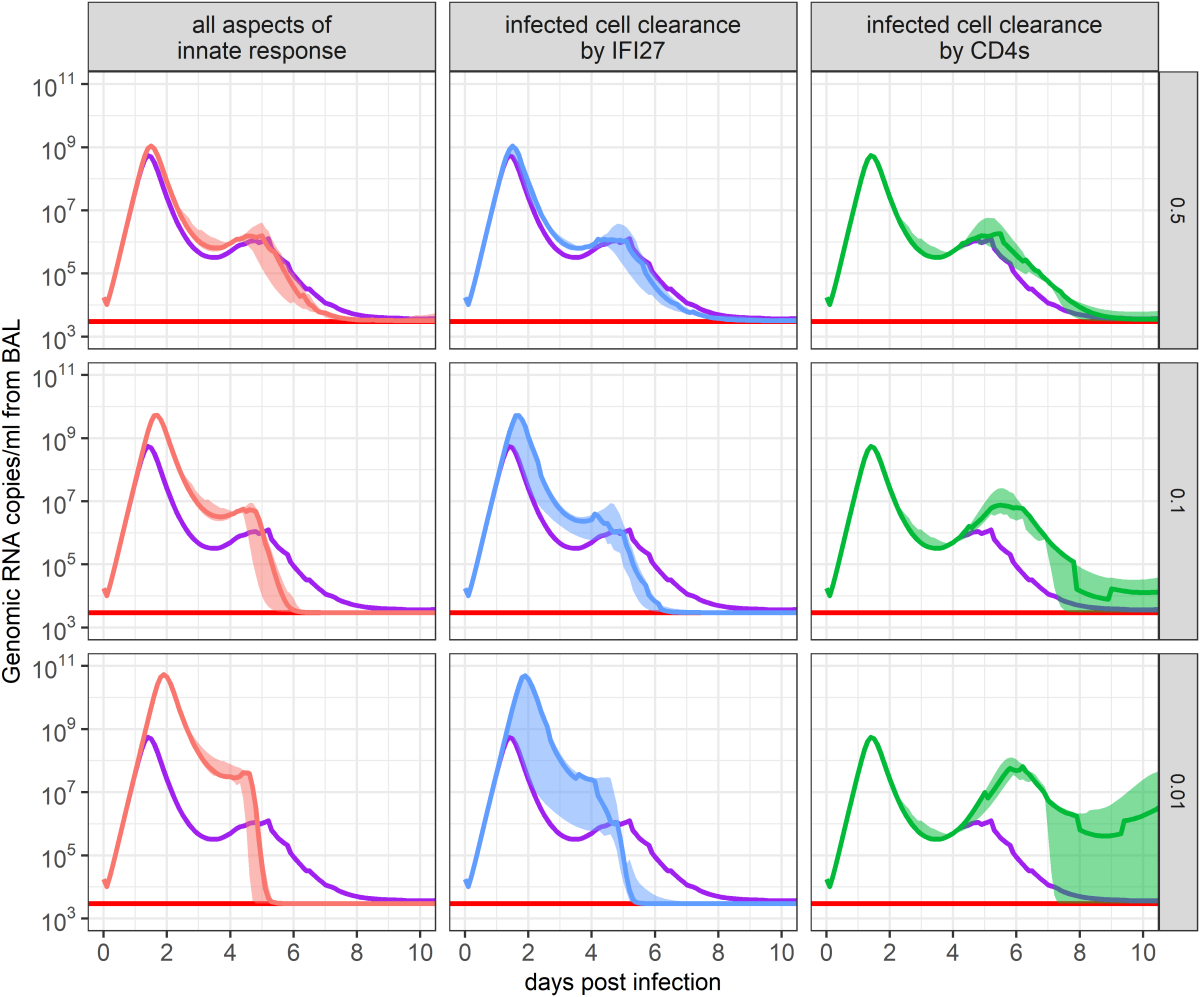
Impact of immune response parameters on the ensemble model’s SARS-CoV-2 viral load projections. The 95% confidence set of best-ranked models was run where each immune response parameter was multiplied by a scaling factor to maintain or dampen its impact on infection. The resulting ensemble weighted median and IQR of predicted viral loads are shown. The scaled parameters descriptions are in the x-axis strip text while the scaling factor is in the y-axis strip text. Red lines show the threshold of detection and purple lines show the weighted median of the unchanged ensemble model. The panel labeled “all aspects of innate response” indicates the impact of all innate immune response parameters ( $b_{Fi}$ and $y_{Fi}$ , where i=1,2,3) appearing in the set of best-ranked models.

To further predict the importance of including each tested term in our mathematical model, we simulated *in silico* knock-down experiments, scaling down model parameter values and observing how these changes impacted predicted viral loads over time. We identified that predicted viral loads showed the greatest increase when we dampened the IFI27-mediated clearance rate of infected cells, regulated by parameter $b_{F1}$ , and the virus-specific CD4+ T cell-mediated clearance rate of infected cells, regulated by parameter n (**Figure 6**).

If parameter $b_{F1}$ was knocked down by 50%, 90%, or 99%, the peak viral load was projected to be 2.0, 9.4, or 89 times higher than in the baseline model, respectively. While viral loads reached higher values in these scenarios, this in turn stimulated more virus-specific CD4+ T cell production due to the greater number of infected cells present (not shown). As a result, there was a faster decline in viral levels once virus-specific CD4+ T cell proliferation began. This is a prediction that should be interpreted with caution because innate regulation also helps induce T cell activation. Similar results occurred when we dampened all innate immune response parameters appearing within the 95% confidence set of models ( $b_{F1},\;b_{F2},\;b_{F3},y_{F1},\;y_{F2}$ and $y_{F3}$ ) (**Figure 6**). This result suggests that IFI27 appears to be the gene best capturing the innate immune response and that its impact on clearing infected cells, rather than dampening infection rates, best described the data.

If parameter n was knocked down, the early viral dynamics remained unaffected due to virus-specific CD4+ T cell proliferation having not yet initiated. Yet, the resurgence of virus predicted at day 4 post-infection was amplified, as the innate response was waning. This outcome is concordant with various degrees of viral rebound noted in untreated individuals in large human cohorts of SARS-CoV-2 infection, particularly during the pre-vaccine era (33). When parameter n was dampened by 50%, 90%, or 99%, viral loads were predicted to rebound to $1.8\times 10^{6}$ , $7.7\times 10^{6}$ , or $6.5\times 10^{7}$ copies/ml of BALF between days 5 and 6 post-infection, respectively. Dampening of other immune parameters or the removal of target cell limitation led to minimal changes in the predicted viral loads of our ensemble model (**Supplementary Material**, **Supplementary Figure S5**), suggesting they have a lesser role in hindering or clearing infection in immunologically naïve animals.

### 2.6 Model forecasts of viral load trajectories given varying degrees of pre-existing innate and acquired immunity

All model fitting was performed on data from previously uninfected and unvaccinated rhesus macaques. The animals therefore had no pre-existing immune memory to SARS-CoV-2. Using our 95% confidence set of models, we attempted to forecast the viral dynamics of re-infection scenarios in rhesus macaques and to assess whether the ensemble model made similar predictions to individual models.

We assumed the same viral inoculum as in the above data but assumed that CD4+ and CD8+ T cell memory would be present due to prior infection, and thus the response to infection by virus-specific T cells would begin sooner. Given the uncertainty regarding the speed of antigen recognition and presentation during early infection, we simulated different scenarios where the CD4+ and CD8+ T cell response was assumed to begin proliferating between 1 and 5 days post-infection (70, 71). Similarly, given the long estimated half-life of memory CD4+ and CD8+ T cells of 200 days (4), we varied the number of virus-specific T cells present in the BALF from values similar to those observed immediately following infection clearance ( ≅65 cells/ml from BAL) to what might be observed 2 years after initial infection ( ≅5 cells/ml from BAL).

**Figure 7:**
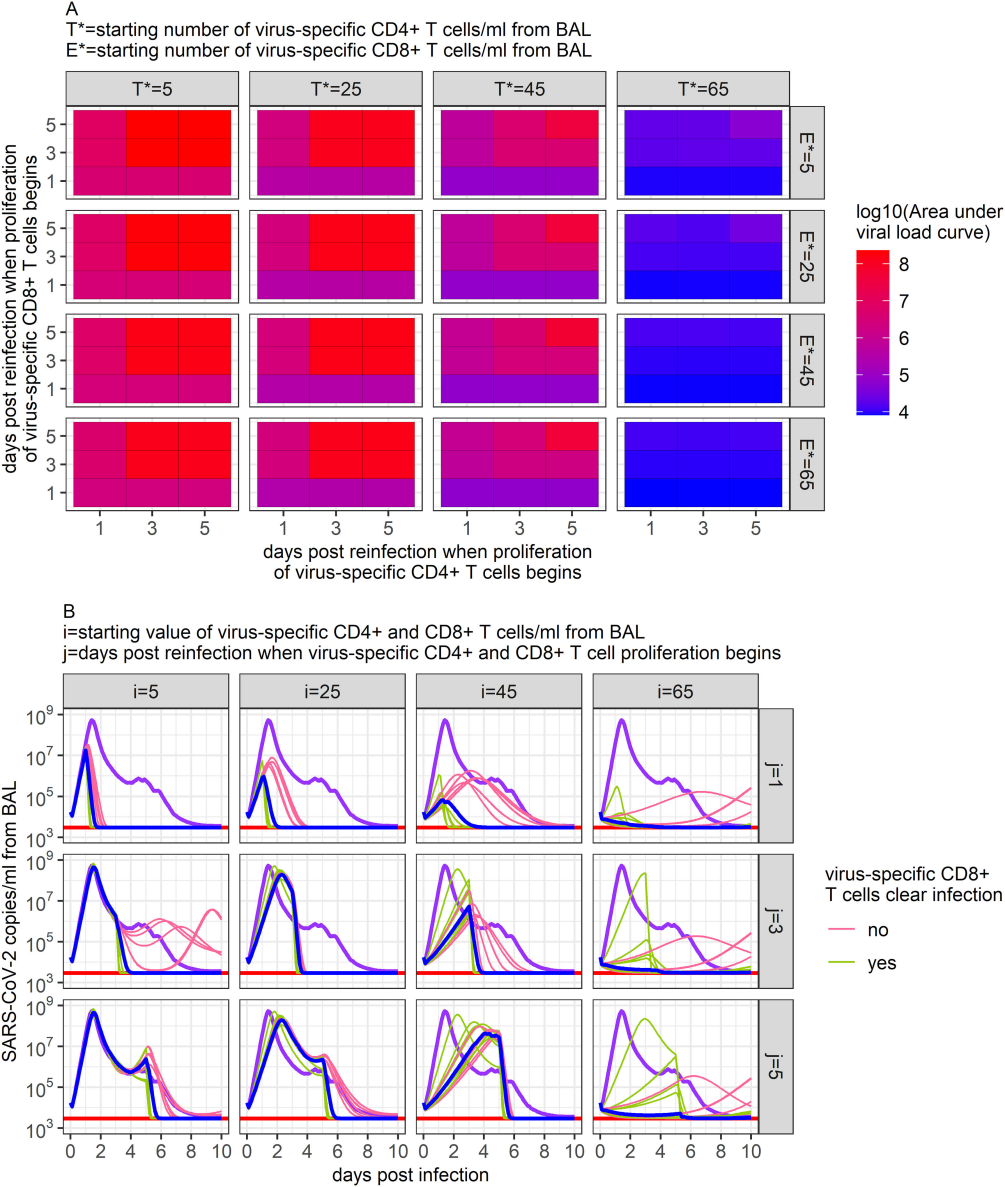
Predictions of SARS-CoV-2 infection viral dynamics assuming different memory T cell conditions. Here, we varied the initial number of virus-specific CD4+ T cells present in the BALF (i) and the time at which memory T cell proliferation was assumed to begin (j) to capture potential conditions during a SARS-CoV-2 reinfection. **(A)** Ensemble model’s predicted peak SARS-CoV-2 viral load (copies/ml) measured from BAL from all scenarios examined. **(B)** Time series dynamics of predicted viral loads from a subset of the scenarios examined (ratio of virus-specific CD4+ T cells to virus-specific CD8+ T cells is 1:1). Purple lines show the ensemble model’s prediction of the viral load during primary infection. Pink and green lines show the individual predictions of the top 12 models within the 95% confidence set. Blue lines show the ensemble model’s predictions of the viral load under each reinfection scenario.

Example results from these simulations are shown in **Figure 7**. Our ensemble model indicates that the number of virus-specific CD4+ T cells present at the start of re-infection has a larger impact on the predicted maximum viral load than the number of virus-specific CD8+ T cells (**Figure 7A**). Using general linear models to predict the relationship between peak viral load and virus-specific CD4+ and CD8+ T cells, we found that while 17 additional virus-specific CD4+ T cells/ml of BALF present at the beginning of infection would lower the peak viral load ten-fold, 450 additional virus-specific CD8+ T cells/ml of BALF would be needed at the beginning of infection to have the same effect. The timing of when virus-specific CD4+ and CD8+ T cells were assumed to begin proliferation positively correlated with the predicted maximum viral load, where each additional delay of a day would increase the maximum viral load by approximately 1/3 of a log.

Despite these trends and the similar dynamics predicted by each of the models within the 95% confidence set when describing primary infection (**Figure 3**), the results of these individual models vary substantially in some of the reinfection scenarios simulated (**Figure 7B**). While all models in the 95% confidence set include infection clearance by virus-specific CD4+ T cells, only half include infection clearance by virus-specific CD8+ T cells. Thus, while increases in virus-specific CD4+ T cells lead to declines in viral load in all models, increases in virus-specific CD8+ T cells only impact viral loads in the models that include their role in infection clearance. This diversion in model predictions is most apparent when the number of virus-specific CD4+ or CD8+ T cells is high (**Figure 7B**) and points to the need for greater model specification against larger and more granular similar datasets, as well as predictive validation against data from animals with previous vaccination and/or infection. Notably, the results from these simulations generate a wide diversity of viral load trajectories with variable peak viral loads, duration of shedding, time to peak, expansion slopes, clearance slopes, and rebound kinetics, all in keeping with results from large human cohorts in the post-vaccination era (33). Overall, these results highlight a potential key impact of pre-infection local immunity on determining the outcome of viral infections.

## 3 Materials and methods

### 3.1 Rhesus macaque infection and data collection

Six healthy male rhesus macaques were infected with $2\times 10^{6}$ TCID_50_ via a combined intranasal and intratracheal inoculation with the USA-WA1 strain of SARS-CoV-2 as described in (2). Infected rhesus macaques were monitored for ten days, with throat, nasal, BALF, and plasma samples collected throughout. BALF samples were used to perform qPCR, cell staining, flow cytometry, ELISA, and scRNAseq as described in (2). All animal experiments and data collection were performed by Nelson et al. (2) at the National Institutes of Health in Bethesda, MD. The single cell RNA sequencing read data can be found at NCBI GEO under accession GSE196980. All other source data and detailed methods for data collection can be found at Nelson et al. (2).

### 3.2 Determining genes of interest based on scRNAseq data

Daily counts/cell for each IFN and ISG examined were averaged across monkeys and then z-score normalized. These values were used in feature selection following the algorithm outlined in (59) and executed using the FSHMM package in R, ranking genes based on their degree of variability over time.

Daily counts/cell were also correlated with the corresponding log_10_-transformed genomic RNA measure made on the same day in the same monkey, as shown in **Figure 1**.

### 3.3 Simulating and fitting our mathematical model

Our mathematical models were simulated and fit using the POMP package in R (72), with a description of this method appearing in the **Supplementary Material**. Models were fit to data presented in **Figure 1** that were transformed to be representative of counts within BALF. A full explanation of how data was transformed and how initial parameter values were chosen appears in the **Supplementary Material**. Models were fit by searching parameter space to maximize each model’s likelihood using the Nelder-Mead algorithm.

The models were concurrently fit to all rhesus macaque data to give maximum power, with a full description of the fitting process in the **Supplementary Material**. Code describing how the model was defined and fit can be found on GitHub at https://github.com/catherinebyrne/SARS-CoV-2-ensemble-model.

### 3.4 Multi-model inference calculations

We compared our different mathematical models with metrics based on AIC scores to better quantify the probability that each model best represented biological data. We calculated the $\text{Δ}AIC_{j}$ for each model i , where


$$\text{Δ}AIC_{j}=AIC_{j}-AIC_{min}$$

and $AIC_{min}$ is the minimum AIC score across all models. From these, we calculated the evidence ratio ( $ER_{j}$ ) of each model which quantifies how much more likely the model with the lowest AIC score is to model j , and is defined as


$$ER_{j}=\frac{e^{-\frac{1}{2}\text{Δ}AIC_{min}}}{e^{-\frac{1}{2}\text{Δ}AIC_{j}}}.$$

Lastly, we calculated AIC weights, where the weight of model j ( $w_{j}$ ) is defined as


$$w_{j}=\frac{e^{-\frac{1}{2}\text{Δ}AIC_{j}}}{\sum_{g=1}^{G}e^{-\frac{1}{2}\text{Δ}AIC_{g}}}$$

and G is the total number of models. These weights represent the probability that a particular model is the best-fitting model of a set, each taking a value between 0 and 1 and summing up to 1.

## 4 Discussion

The ever-growing volume and complexity of non-linear immunologic data from animal model infection experiments and from human research protocols underscores the necessity of developing new computational methods for transforming these data from being descriptive to more mechanistic. Many SARS-CoV-2 datasets would benefit from such methods, as the data’s multidimensionality poses substantial challenges in discerning the precise components of the innate and adaptive immune response driving viral elimination, and immunopathogenesis.

While mechanistic mathematical modeling can be highly beneficial to gain insight into the non-linear dynamics and interplay between pathogens and the immune response (30–33), how to appropriately scale up these models to incorporate multiple immune data types is largely unestablished. In many fields, like climate/weather modeling, ecology, and epidemiology, many models are often created, compared for likelihood, and ultimately combined into an ensemble model, allowing for a more realistic and unbiased approach to forming predictions and understanding a system (51–57). However, this technique has yet to be widely applied to within-host mathematical models of infectious disease. Through this approach, we attempted to fit 160 different mathematical models to data describing SARS-CoV-2 infection within the lungs of rhesus macaques, with each model encoding a specific set of hypotheses describing how the immune response may target and clear SARS-CoV-2 infection.

Through feature selection and correlation of IFN and ISG expression with viral load, we identified IFI27, IFI6, and IFI16 as innate genes likely linked to SARS-CoV-2 clearance and ones whose dynamics may be representative of the entire innate immune response. These genes were also selected based on experimental evidence from human infection linking their expression to viral clearance, though conflicting data from mouse models emphasizes the need to test our model’s conclusions further (11, 60, 69, 73). Upon fitting our models to these data, IFI27 emerged as the gene best capturing the innate immune response against SARS-CoV-2. Previous studies have identified the IFI27 gene to be one that is upregulated in the lung tissue of SARS-CoV-2 patients (74), and where expression temporally aligns with SARS-CoV-2 viral loads (11). IFI27 is suggested to regulate the innate immune response during SARS-CoV-2 infection by interfering with the RIG-1 pathway, which is critical for recognizing viral infection and preventing hyper-inflammation and excessive innate immune responses (60, 73). Through analysis of our top-ranked models, the proliferation of the innate immune response was suggested to largely limit infection through direct clearance of infected cells.

Analysis of our different mathematical models also indicated that virus-specific CD4+ T cell-mediated clearance of infection had high importance in capturing infection dynamics, appearing in all top-ranked models. Similarly, clearance of infection by virus-specific CD8+ T cells appeared in half of top-ranked models. Previous research has highlighted the importance of the T cell response in clearing and mitigating the severity of SARS-CoV-2 infection, with the cytotoxic role of CD8+ T cells well-established (42, 75–77). This feature of infection may have been underestimated in our model based on the inclusion of only immunologically naïve animals and highlights the importance of future work fitting models concurrently to infected immune experienced animals as well. While CD4+ T cells are usually thought to help clear infection indirectly through activating and recruiting other immune cells, cytokine production, and regulating the immune response, some studies of SARS-CoV-2 infection have reported significant expansion of cytotoxic CD4+ T cells within infected patients, particularly in those with severe COVID-19 (78–80). Thus, the importance of virus-specific CD4+ T cell-mediated clearance of infection within our model may be a result of its help to other immune cell populations, or through direct cytotoxic activity.

Our model suggested that anti-spike IgG antibodies do not play a discernable role in infection clearance within the first 10 days of infection in immune-naïve animals. Anti-spike IgG titration curves showed no significant change over the 10 days of study (2), and previous studies suggest that titers do not peak until 3 to 7 weeks post-infection (81). While much literature describes the importance of the antibody response against SARS-CoV-2 (22, 81, 82), these responses may instead play more of a role in preventing and controlling re-infection. Indeed, previous mathematical analysis has also shown that plasma SARS-CoV-2 antibodies likely do not impact primary infection, with increases in titers not occurring until later post-infection (35).

Through simulating our top-ranked models when assuming the presence of T cell memory, our results suggest how memory T cells may limit the severity of reinfection, with virus-specific CD4+ T cells having a larger impact than virus-specific CD8+ T cells. While all top-ranked models performed similarly when capturing available primary infection data, they notably diverged in their forecasts of infection when large populations of memory T cells were assumed to be present. By using an ensemble model approach, our predictions consider these differences and describe the uncertainty associated with projections in a way that a single-model analysis would not. This result underscores the importance of ensemble model techniques, especially when forming predictions beyond what available data describes.

The uncertainty of our predictions could be narrowed if our ensemble model reflected the input of fewer models, if top-ranked models differed less substantially in terms of assumptions, and if the top-ranked models had a higher relative likelihood of explaining the data. To achieve this aim would require fitting models to a wider array of strategically gathered data. In current work, this could be achieved by increasing the amount and type of viral and immune data with a possible focus on gathering these measures at least daily. A common problem for model fitting is that longitudinal sampling is too infrequent. However, in our case, more data time points describing early primary infection may or may not have narrowed the scope of possible best models, as all our top-ranked models predicted similar dynamics during the first 10 days of primary infection. Instead, further testing our models with data collected from other infection scenarios, such as reinfection, or infection following vaccination might effectively rule out the likelihood of multiple models within our current 95% confidence set. These different scenarios may also better reveal the influence of cellular and humoral immunity, which may play a more critical role in repeated infections than our top models indicate.

Another limitation of our models is that they do not include the inherent spatial, multi-compartment dynamics of infection. Our mathematical models are meant to be representative of the infection kinetics within the lung, with the data used to fit our models measured from BALF. While BALF does provide some representation of the immune and viral changes within the secretions of the alveoli of the lower respiratory system, its sampling does not directly capture what is occurring within the lung’s mucosa, where infected cells would be present, and tissue-resident immunity may reside. Further, our model treats the lung as a homogeneous region; instead, patches of infected cells and a heterogeneous immune response across different tissue regions are likely present.

Another possible pitfall is subjectivity in terms of model design such that the most likely model may not be included in the list of considered models. Similarly, if key immune mediators of viral clearance are not measured and used for model fit, then these mechanisms will inherently be underrepresented in the ensemble model. These problems are not unique to ensemble models and are relevant to more traditional viral dynamic models as well as immunologic experiments in general. Nevertheless, future work will need to strive to achieve a balance between comprehensive sampling of all arms of the immune response to avoid biasing model conclusions.

Our study demonstrates the potential of integrating diverse mathematical models and ensemble techniques to dissect the dynamics of viral clearance and immune response. These methods will become increasingly necessary due to the breadth and complexity of immune data being collected during longitudinal infection studies. By identifying key genes and predicting the timing and way in which the innate and adaptive immune systems each respond to SARS-CoV-2 infection, our results provide early insights into the intricate interplay between SARS-CoV-2 and the host immune system, while highlighting the need for careful experimental design to best inform these models.

## Data Availability

The datasets presented in this study can be found in online repositories. The names of the repository/repositories and accession number(s) can be found in the article/**Supplementary Material**.
