# Supplementary material for: Ensemble modeling of SARS-CoV-2 immune dynamics in immunologically naïve rhesus macaques predicts that potent, early innate immune responses drive viral elimination
Source: Front Immunol. 2024 Nov 7;15:1426016. doi: 10.3389/fimmu.2024.1426016 (PMC11578959; doi:10.3389/fimmu.2024.1426016)
Supplement: Supplementary file 1 [file DataSheet1.pdf]

Supplementary Material

1 Converting available data to be representative of counts within BALF

While a wide breadth of data was generated to characterize the viral and immune response kinetics within the lung of SARS-CoV-2 infected rhesus macaques through BALF sampling, not all data were immediately in a form representative of "counts". As our mathematical models describe the time evolution of the counts for each compartment modeled, we were required to apply some assumptions and transformations to the data to make it so.

1.1 Viral load

SARS-CoV-2 genomic RNA was measured in units of copies per ml of BALF, which we assumed to be representative of the number of infectious SARS-CoV-2 virions within the lung. As the method for sampling of BALF remained constant throughout the study and was presented as a count, we directly fit this data without any transformation. However, while SARS-CoV-2 gRNA was measured on days 1, 2, 4, 7, and 10 post-infection in the throat, nose, and plasma, it was only measured on days 4, 7, and 10 in BALF (1). While gRNA loads within plasma remained below the level of detection in all samples apart from one and did not appear similar to what was seen in the BALF (1), gRNA within the BALF, nose, and throat followed a similar trajectory over time (Figure S. 1). gRNA loads within the BALF on days 1 and 2 post-infection were thus extrapolated by developing a linear model in R where the ln of BALF gRNA was assumed to be a function of the ln of nose and ln of throat measures, ensuring the model passed through the point (0,0,0). Resulting data points are shown in Figure 1 of the main paper.

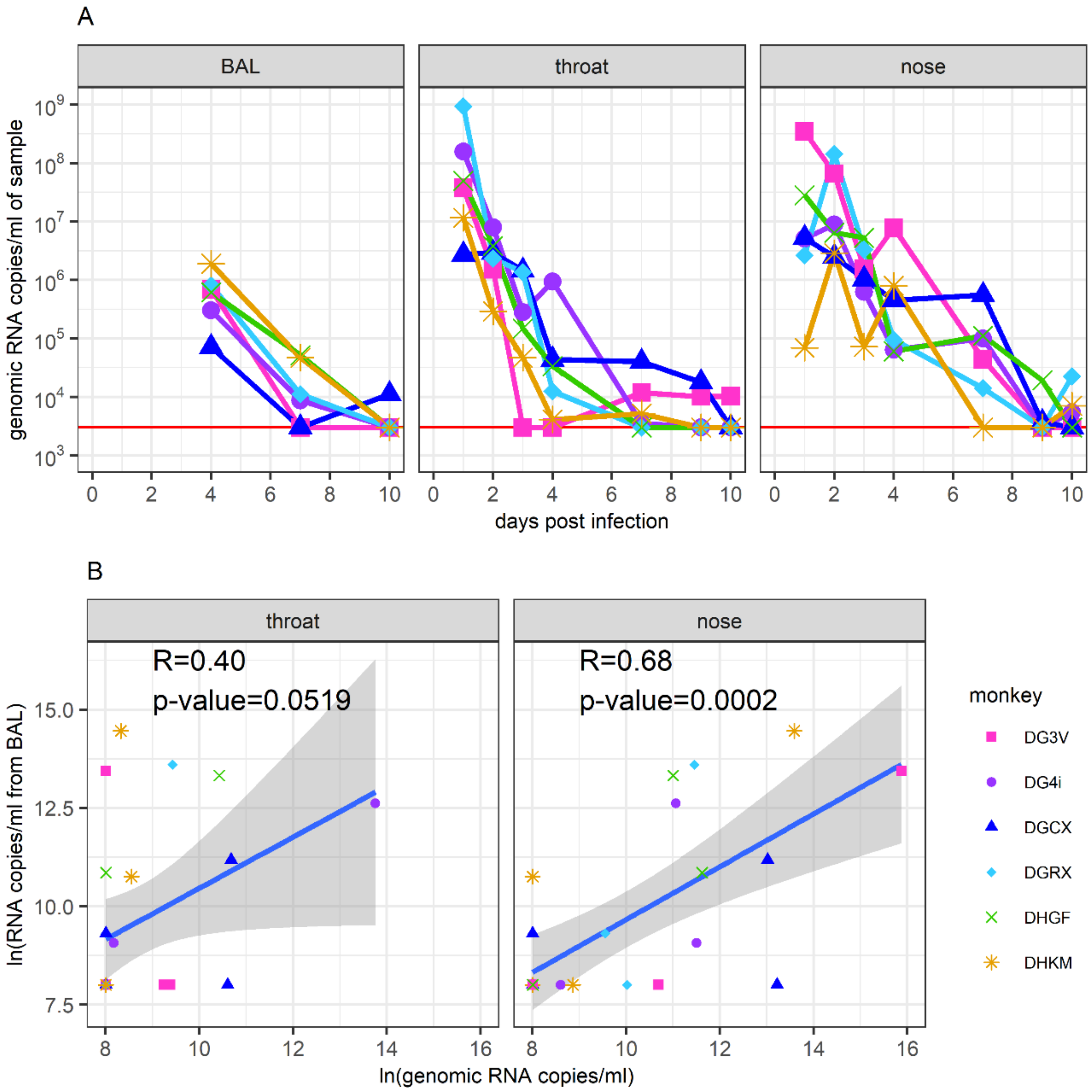

Figure S. 1: Relationship between gRNA loads seen in BALF, throat, and nose samples. Panel A shows the genomic RNA copies/ml found in BALF, throat, and nose samples over the course of observation. Panel B shows the correlation between gRNA loads in BALF samples and in throat, and nose samples. Using these relationships, a linear model to predict the gRNA loads in BALF samples as a function of throat and nose sample loads was calculated. This equation was used to predict BALF gRNA loads on days 1 and 2 post-infection.

1.2 Expression of ISGs

We assumed that the expression of ISGs that were determined to be of interest (Methods) was representative of the innate immune response. The expression of ISGs within BALF is reported in units of copies/cell (1). As we did not have access to any data indicating the change in the number of cells within BALF over the course of infection, we assumed this to be a constant. As such, we treated gene expression values as counts and used these values directly when fitting our mathematical models.

1.3 Anti-spike IgG antibody

Anti-spike IgG was measured in BALF using fluorescent ELISA and reported in units of AUC for titration curves, where the relative fluorescent units measured were integrated over the different dilution factors measured (1). Generally, as in this scenario, when standards are not available to be used in ELISA experiments to directly determine the antibody concentrations of samples, AUC is a common metric for analyzing antibody assays (2). As concentrations of anti-spike IgG within BALF could not be directly determined here, we assumed a linear relationship between AUC measures and antibody concentrations and thus treated AUC measures as a count when fitting our mathematical models.

1.4 Virus-specific CD4+ and CD8+ T cells

The flow cytometry data from (1) provide a wealth of information on the change in different immune cell populations over the first 10 days of SARS-CoV-2 infection. In particular, the presence of virus-specific CD8+ and virus-specific CD4+ cells was determined by stimulating BALF cells with a super pool of SARS-CoV-2 peptides and staining for IFNγ or TNF, with the expression of either indicating a cell was virus-specific (1). While data describing the number of virus-specific CD4+ and CD8+ cells/ml of BALF over time would be ideal for our mathematical models of infection, data instead presents the % virus-specific of CD8+ cells and the % virus-specific of CD4+ cells. To make these data conducive to modeling, we converted these values into counts/ml of BALF.

In addition to % virus-specific of CD8+ cells and % virus-specific of CD4+ cells, (1) also reported the % CD4+ of CD3+ cells, the % CD4+ of CD3+ cells, and the % CD3+ of live cells in rhesus macaques during SARS-CoV-2 infection.

Using these data and knowing that

$$\frac{\%\ \mathrm{CD8}^{+}\ \text{of}\ \mathrm{CD3}^{+}\ \text{cells}}{100}\cdot\frac{\%\ \mathrm{CD8}^{+}\ \text{of live cells}}{100}\cdot 100 = \%\ \ \mathrm{CD8}^{+}\ \text{of live cells}$$

and

$$\frac{\%\ \ \mathrm{CD4}^{+}\ \text{of}\ \mathrm{CD3}^{+}\ \text{cells}}{100}\cdot\frac{\%\ \mathrm{CD3}^{+}\ \text{of live cells}}{100}\cdot 100 = \%\ \mathrm{CD4}^{+}\ \text{of live cells}$$

we found that before infection the rhesus macaques in this experiment had an average of 30.4% CD8+ of live cells in BALF and 22.5% CD4+ of live cells in BALF prior to infection.

While we could not find estimates of the total number of CD8+ or CD4+ cells in the BALF of healthy monkeys, (3) estimated there to be 1100 CD8+ cells/ml of BALF in humans. Assuming that the number of CD8+ cells /ml in BALF is the same in healthy humans and rhesus macaques, we thus assumed there to be 1100 $\mathrm{CD8}^{+}$ live cells/ml of BALF in rhesus macaques. Furthermore, using the above calculated % CD8+ of live cells and % CD4+ of live cells, the number of CD4+ live cells/ml of BALF can be calculated as

$$1100\ \text{CD8}^{+}\ \text{live cells/ml} \cdot \frac{22.5\%\ \text{CD4}^{+}\ \text{of live cells}}{30.4\%\ \text{CD8}^{+}\ \text{of live cells}}$$

$$= 814\ \text{CD4}^{+}\ \text{live cells/ml}$$

Using these counts/ml, we can now convert the % virus-specific of CD8+ cells and % virus-specific of CD4+ cells data available from (1) to counts/ml for modeling.

Letting the total CD8+ cell count/ml at time t be termed $E_{all}(t)$, the virus-specific-CD8+ T cell count/ml be termed $E(t)$ and the non-virus-specific CD8+ T cell count/ml be termed $E_N(t)$, we know that at any time

$$E_{all}(t) = E(t) + E_N(t).$$

Using the % virus-specific of CD8+ cells, termed $P_E(t)$ here, $E(t)$ can also be written as

$$E(t) = P_E(t)/100 \cdot E_{all}(t)$$

Using the above two equations and solving for $E(t)$ we get

$$E(t) = E_N(t) \cdot \frac{P_E(t)/100}{1 - P_E(t)/100}.$$

We now make the assumption that at $t = 0$, there is a negligible number of virus-specific CD8+ cells, and thus $E_N(0) = 1100$ non-virus-specific CD8+ live cells/ml. We further make the assumption that the proliferation of $E_N(t)$ is negligible over the course of infection and thus remains a constant. While this is likely untrue, it provides a conservative estimate for the total number of virus-specific CD8+ T cells/ml during infection.

We perform similar calculations to determine the number of virus-specific CD4+ T cells/ml from available data, again assuming there is a negligible number of virus-specific CD4+ T at $t = 0$ and that the proliferation of non-virus-specific CD4+ live cells was negligible. Table S. 1 displays both the percentages of virus-specific CD4+ and CD8+ T cells taken from (1) and our calculated counts over the course of infection (rounded to the closest whole cell) that we used when fitting our mathematical model.

Table S. 1: Conversion of virus-specific CD4+ and CD8+ T cell measurements into count data. The Percent virus-specific of CD4+ and CD8+ cells was converted into counts of virus-specific CD4+ and CD8+ cells as described above.

Days post infection	Monkey ID	% virus-specific of CD8+ cells	# virus-specific CD8+ cells/ml BALF	% virus-specific of CD4+ cells	# virus-specific CD4+ cells/ml BALF
0	DGRX	0	0	0	0
4	DGRX	0	0	0.06	0
7	DGRX	0	0	11.94	110
10	DGRX	0.79	9	5.86	51
0	DG4i	0	0	0.46	4
4	DG4i	0	0	0	0
7	DG4i	0.05	1	7.38	65
10	DG4i	2.45	28	8.59	76
0	DGCX	0	0	0.17	1
4	DGCX	0	0	0	0
7	DGCX	0	0	2.22	18
10	DGCX	5.75	67	3.48	29
0	DG3V	0	0	0.05	0
4	DG3V	0	0	0	0
7	DG3V	1.21	13	3.46	29
10	DG3V	17.93	240	8.54	76
0	DHGF	0	0	0	0
4	DHGF	0.17	2	0.02	0
7	DHGF	0.96	11	2.93	25
10	DHGF	4.39	51	1.64	14
0	DHKM	0.09	1	0.13	1
4	DHKM	0	0	0.03	0
7	DHKM	1.92	22	6.84	60
10	DHKM	3.6	41	3.66	31

2 Fitting data and simulating models using the pomp package in R

We used the R pomp package, developed by Aaron King (4), to write, fit and simulate our models. While the pomp package is specifically designed to describe non-linear partially observed Markov processes, it generally provides a flexible framework for describing and fitting many systems of ordinary differential equations of varying structure. The pomp package takes a likelihood-based approach when fitting a complex model, allowing for the user to define custom likelihood functions that describe how the observed data relates to the states of the model (4). With this function, one can evaluate how well the model predicts the observed data, given a certain set of parameter values.

Here, we defined the likelihood function as the sum of the probability of each data point given the model prediction, where the observation of ISG expression, virus-specific CD4+ T cells, and virus-specific CD8+ T cells were assumed to be drawn from a Poisson distribution, and the log-value of gRNA and anti-spike IgG titers were assumed to be drawn from a Normal distribution. In

conjunction with the Nelder Mead algorithm for searching parameter space (5), the parameter values that maximize this likelihood function were found.

Initial conditions for the number of susceptible lung epithelial cells were set to 1.645×10^9, based on (6). As the rhesus macaques used in this study had never been previously exposed to SARS-CoV-2, the initial number of virus-specific CD4+ and CD8+ T cells were both set to 0, matching with what was observed in the data set (Figure 1 of the main paper). The initial number of IFI27, IFI6, and IFI16 ISG copies/cell were set to the median baseline value recorded in the data set across monkeys. The initial number of SARS-CoV-2 copies/ml from BAL and the initial AUC for IgG anti-spike titration curves from BAL were determined by an initial parameter search to optimize simulations; these values were then fixed for the remainder of all model fitting. All other parameter values were allowed to vary during model fitting. Upon completing model fitting, alternate initial values of AUC for IgG anti-spike titration curves from BAL were found to lead to worst fits. Alternate initial numbers of SARS-CoV-2 could be used to potentially explain the data; however, they required compensation through changes in other virus-related parameters, leading to identifiability issues if done.

Fitting of each model was performed 4 times, each with different starting parameter values for those that were allowed to vary. For each of the 4 rounds of fitting, the Nelder-Mead algorithm was repeated multiple times until the likelihood value converged, with the likelihood value of the last run being less than 0.5 units better than the likelihood value of the previous run. After the 4 rounds of fitting, the AIC value from each run was calculated for each model. The lowest AIC value for each model was used to determine the final ranking of models, as shown in Figure S.2. and Figure 3 of the main paper. The variability in AIC values from these 4 rounds of fitting is shown in Figure S.2, and generally was low. The set of parameter values that led to each model's lowest AIC value was also considered the final, best fitting set for that model and was used when examining the parameter distributions displayed in Figure 4 of the main paper. A description of all parameter values and their initial starting conditions appears in Table S.2.

Table S. 2: Definitions of Model Parameters. The definition of each model parameter included within our model appears here. The initial starting value for the first of the four rounds of parameter fitting is shown. For the other 3 rounds, the initial starting value was randomly selected to lie within the range of 10% less to 10% greater of the value listed. Parameters marked with a * were not varied during fitting and remained fixed at the starting value listed. When simulating the different versions of the base model we developed (Figure 2 of the main paper), some terms and parameters were not always included. These are indicated here.

Parameter	Description	Starting Values	Units
d_S	Per-capita death rate of susceptible cells	2.90×10^{-2}	Per susceptible cell per day
η	Maximum per-virion infection rate of susceptible cells	1.82×10^{-2}	Per gRNA copy/ml per day
y_A	Antibody's rate of impact on dampening the infection rate of susceptible cells by virions	1.0×10^{-6} if including in model	Per unit of AUC for anti-spike titration curve
δ	Per-capita natural death rate of infected cells	2.37	Per infected cell

α_E	Per-capita rate at which each infected cell leads to the generation of more virus-specific CD8+ T cells. This term only takes effect once proliferation of virus-specific CD8+ T cells is turned on at $t = \tau_E$.	5.44×10^{-1}	Per infected cell per day
α_T	Per-capita rate at which each infected cell leads to the generation of more virus-specific CD4+ T cells. This term only takes effect once proliferation of infection-induced virus-specific CD4+ T cells is turned on at $t = \tau_T$.	7.15×10^{-3}	Per infected cell per day
α_{F1}	Per-capita rate at which each infected cell leads to the generation of more IFI27. This term only takes effect once infection-induced production of IFI27 is turned on at $t = \tau_{F1}$.	6.17×10^{-5}	Per infected cell per day
α_{F2}	Per-capita rate at which each infected cell leads to the generation of more IFI6. This term only takes effect once infection-induced production of IFI6 is turned on at $t = \tau_{F2}$.	9.90×10^{-5}	Per infected cell per day
α_{F3}	Per-capita rate at which each infected cell leads to the generation of more IFI16. This term only takes effect once infection-induced production of IFI16 is turned on at $t = \tau_{F3}$.	9.90×10^{-5}	Per infected cell per day
α_A	Per-capita rate at which each infected cell leads to the generation of more anti-spike IgG antibody. This term only takes effect once infection-induced production of anti-spike IgG antibody is turned on at $t = \tau_A$.	1.0×10^{-8} if including in model	Per infected cell per day
m	Mass action clearance rate of infected cells attributed to virus-specific CD8+ T cells	0.1 if including in model	Per virus-specific CD8+ T cell/ml per infected cell per day
n	Mass action clearance rate of infected cells attributed to virus-specific CD4+ T cells	0.1 if including in model	Per virus-specific CD4+ T cell/ml per infected cell per day

b_{F1}	Mass action clearance rate of infected cells attributed to IFI27	0.1 if including in model	Per IFI27 gene copy/cell per infected cell per day
b_{F2}	Mass action clearance rate of infected cells attributed to IFI6	0.1 if including in model	Per IFI6 gene copy/cell per infected cell per day
b_{F3}	Mass action clearance rate of infected cells attributed to IFI16	0.1 if including in model	Per IFI16 gene copy/cell per infected cell per day
p	Maximum per-capita production rate of virions by infected cells	2.93×10^4 if including in model	Per infected cell per day
y_{F1}	IFI27's rate of impact on dampening the production rate of virus by infected cells	0.1 if including in model	Per IFI27 gene copy/cell
y_{F2}	IFI6's rate of impact on dampening the production rate of virus by infected cells	0.1 if including in model	Per IFI6 gene copy/cell
y_{F3}	IFI16's rate of impact on dampening the production rate of virus by infected cells	0.1 if including in model	Per IFI16 gene copy/cell
c	Per-capita natural clearance rate of virions	3.67×10^1	Per gRNA copy/ml per day
d_{F1}	Per-capita natural clearance rate of IFI27	1.65×10^{-1}	Per IFI27 gene copy/cell per day
d_{F2}	Per-capita natural clearance rate of IFI6	5.97×10^{-1}	Per IFI6 gene copy/cell per day
d_{F3}	Per-capita natural clearance rate of IFI16	3.70×10^{-1}	Per IFI16 gene copy/cell per day
d_T	Per-capita natural clearance rate of virus-specific CD4+ T cells	3.00×10^{-2}	Per virus-specific CD4+ T cell/ml per day
d_E	Per-capita natural clearance rate of virus-specific CD8+ T cells	1.11×10^{-2}	Per virus-specific CD8+ T cell/ml per day
d_A	Per-capita natural clearance rate of anti-spike IgG antibody	Not fit due to lack of change in this compartment over time	AUC for anti-spike titration curve per day
τ_E	The time at which infection-induced proliferation of virus-specific CD8+ T cells is assumed to begin	6.93	days
τ_T	The time at which infection-induced proliferation of virus-specific CD4+ T cells is assumed to begin	5.41	days

τ_{F1}	The time at which infection-induction IFI27 production is assumed to begin	3.84×10^{-2}	days
τ_{F2}	The time at which infection-induced IFI6 production is assumed to begin	3.84×10^{-2}	days
τ_{F3}	The time at which infection-induced IFI16 production is assumed to begin	3.84×10^{-2}	days
τ_A	The time at which infection-induced anti-spike IgG antibody production is assumed to begin	9	days
$^*T_0$	The initial amount of virus-specific CD4+ T cells	0	Virus-specific CD4+ T cells/ml of BAL
$^*E_0$	The initial amount of virus-specific CD8+ T cells	0	Virus-specific CD8+ T cells/ml of BAL
$^*F1_0$	The initial amount of IFI27	0	RNA copies/cell from BAL
$^*F2_0$	The initial amount of IFI6	3	RNA copies/cell from BAL
$^*F3_0$	The initial amount of IFI16	6	RNA copies/cell from BAL
$^*V_0$	The initial number of virions	1.47×10^4	SARS-CoV-2 gRNA copies/ml from BAL
$^*A_0$	The initial amount of anti-spike IgG antibodies	3.67×10^5	AUC for anti-spike titration curves from BAL
$^*I_0$	The initial number of infected cells	0	Cells in lung
$^*S_0$	Initial number of susceptible cells	1.645×10^{-9} if including in model	Cells in lung

3 Additional Graphs

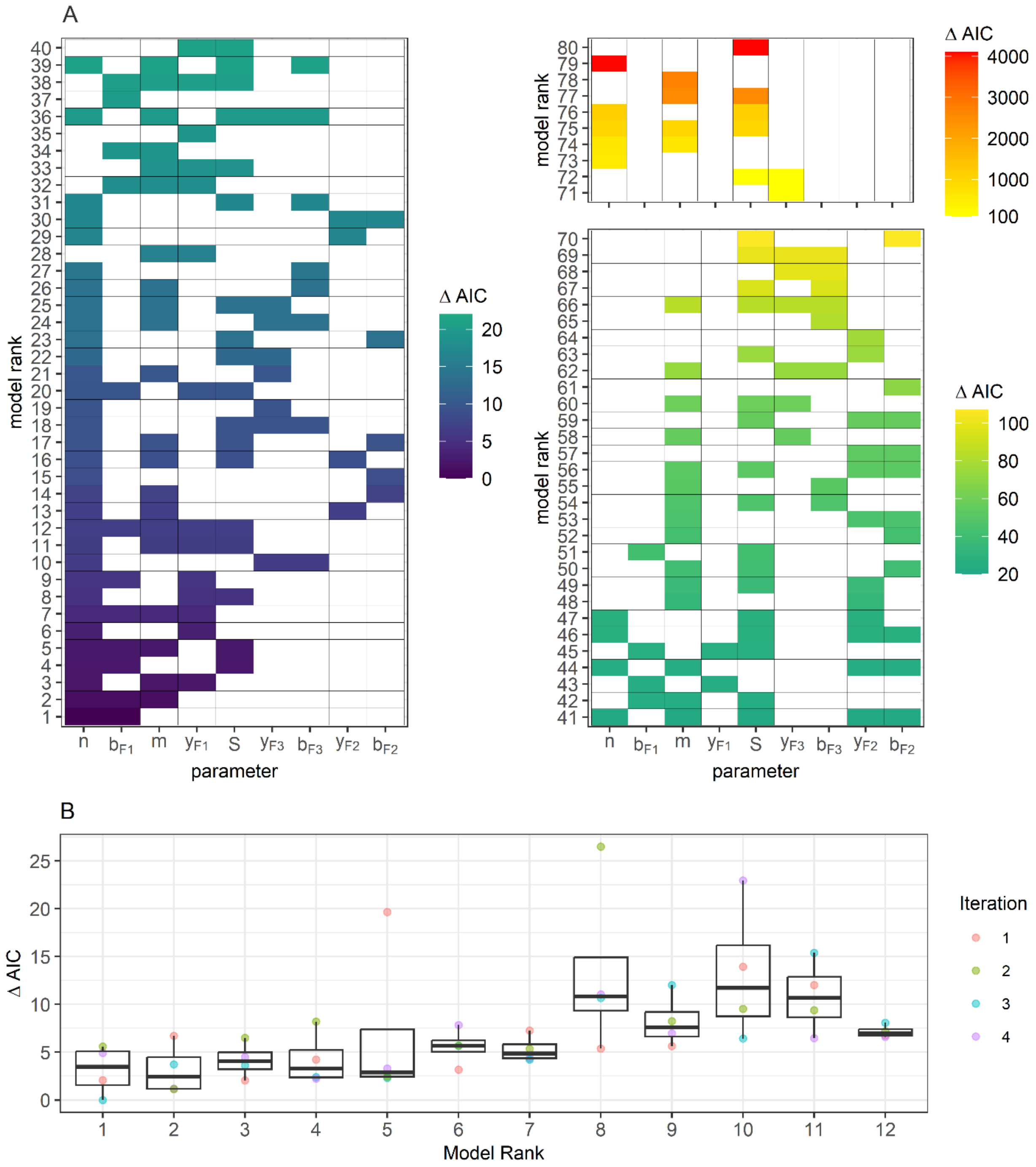

Figure S. 2: Comparing AIC scores from all models and the variance in different rounds of model fitting. Fitting for each potential model was repeated four times. The lowest AIC score for each model was used to determine its final rank. Panel A shows the *Δ*AIC score for the 80 models examined. *Δ*AIC is calculated by subtracting off the top-ranked model's best AIC (1528.5). Models

including the impact of anti-spike IgG are excluded due to its lack of change in size over time. Panel B displays the ΔAIC scores from all four rounds of model fitting for the top 12 models.

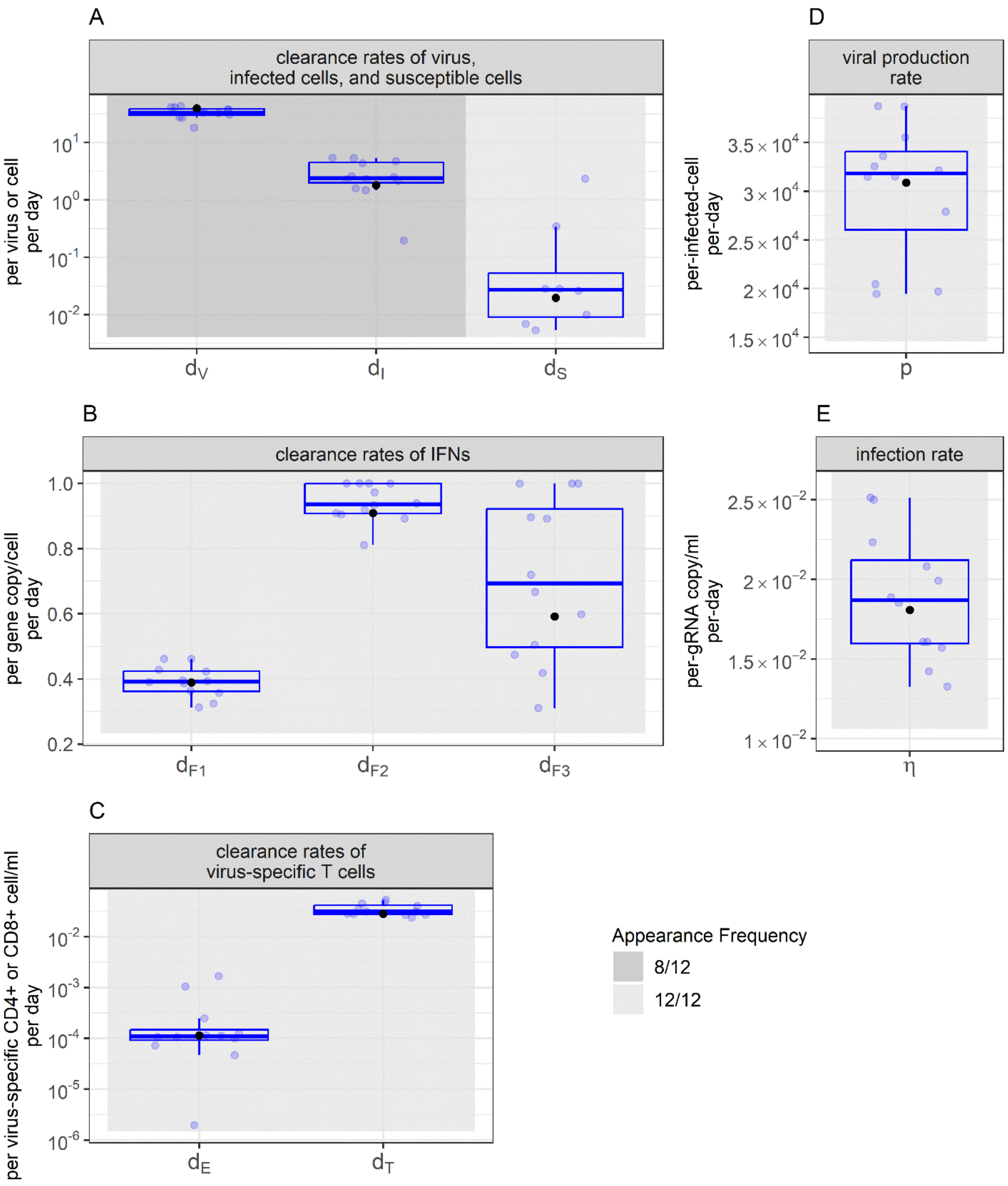

Figure S. 3: Other parameter values appearing for the best-ranked models. Background shade indicates how often a parameter appeared within the 12 best-ranked models. Blue dots show individual parameter values for the models that include each parameter (value not set to 0). Boxes show the median and interquartile range (IQR), while whiskers indicate 1.5 times the IQR. Black

dots indicate the weighted median of each parameter value, determined using model Akaike weights when accounting for all models in the 95% confidence set.

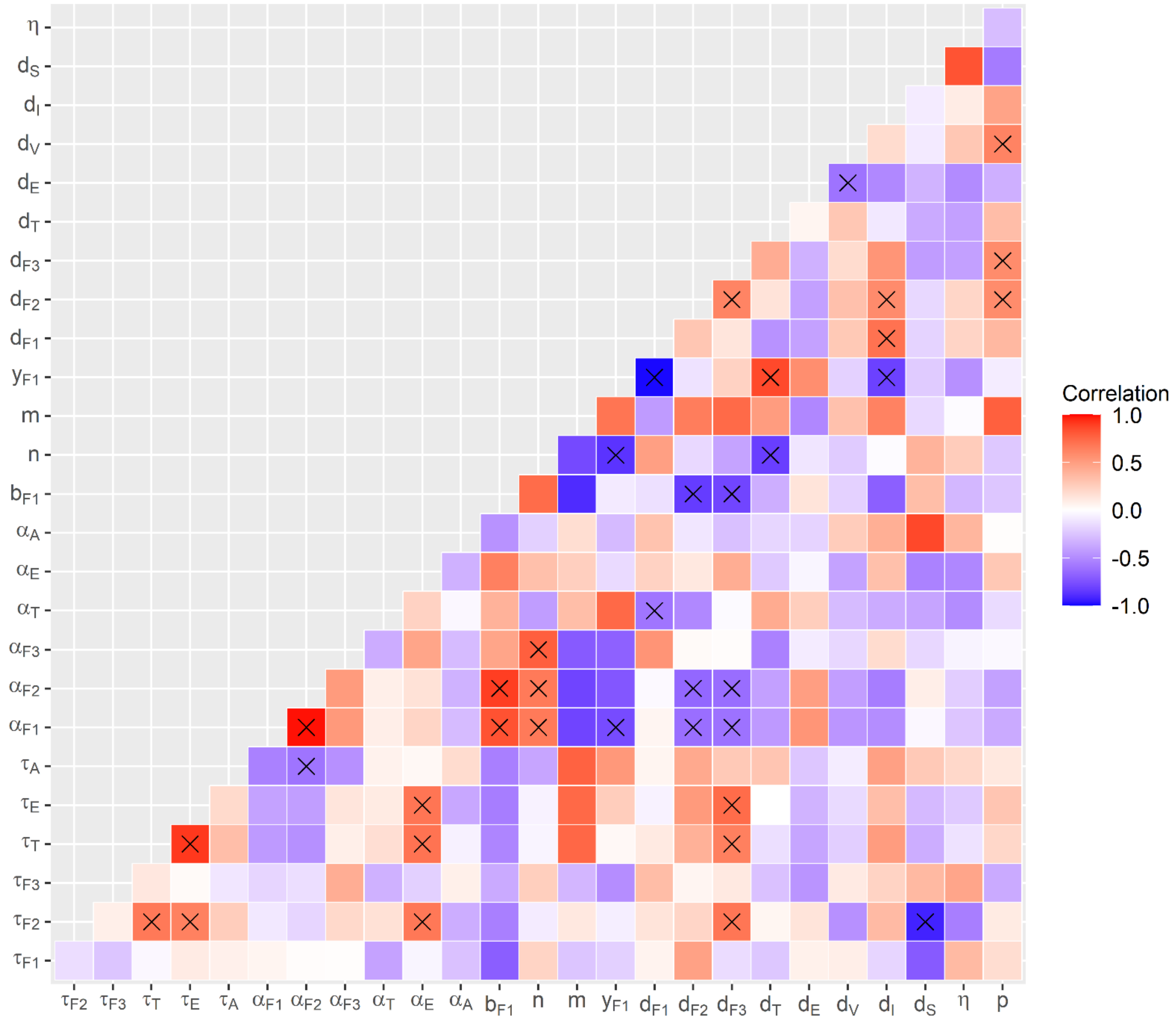

Figure S. 4: Correlation coefficients between parameters appearing within the 95% confidence set of best-ranked models. Colour indicates the correlation coefficient while X's indicate which correlation values are significant, with a p-value<0.05. Parameters that did not appear within the

best-ranked models or did not appear frequently enough to calculate a correlation coefficient have been removed.

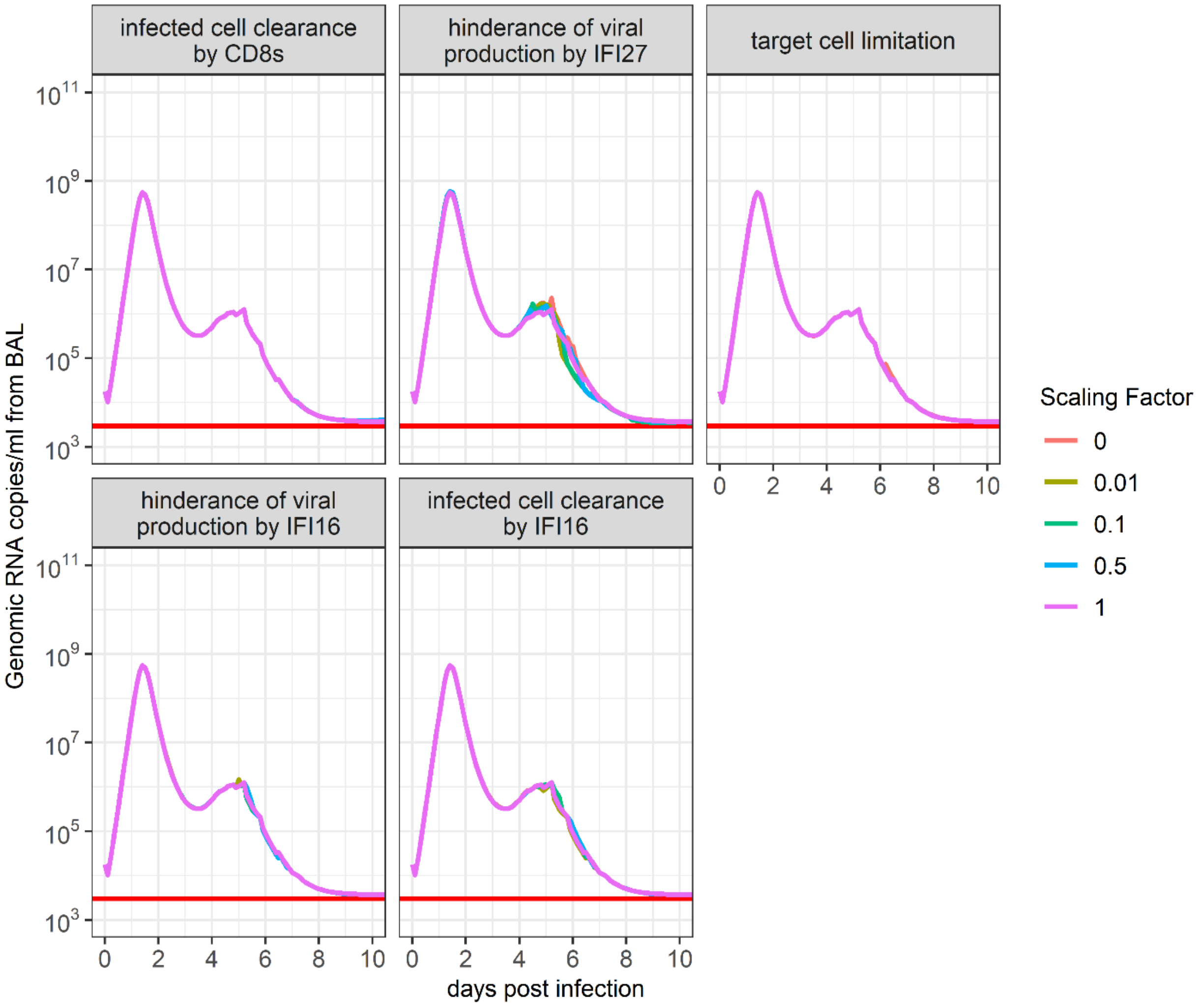

Figure S. 5: Impact of lower-importance immune response parameters on the ensemble model's SARS-CoV-2 viral load projections. The 95% confidence set of best-ranked models was run where each immune response parameter was multiplied by a scaling factor to maintain or dampen its impact on infection. The resulting ensemble weighted median of predicted viral loads is shown. The scaled parameter descriptions are in the x-axis strip text while the scaling factor is indicated by the colour of the lines shown. Note that many of the lines overlap and are undistinguishable from the line where the scaling factor is 1. Red lines indicate the threshold of detection.

4 References

1. Nelson CE, Namasivayam S, Foreman TW, Kauffman KD, Sakai S, Dorosky DE, et al. Mild SARS-CoV-2 infection in rhesus macaques is associated with viral control prior to antigen-specific T cell responses in tissues. Sci Immunol. 2022 Apr 29;7(70):eabo0535.

2. Yu X, Gilbert PB, Hioe CE, Zolla-Pazner S, Self SG. Statistical approaches to analyzing HIV-1 neutralizing antibody assay data. Stat Biopharm Res. 2012 Jan 1;4(1):1–13.

3. Emad A, Emad Y. Increased in CD8 T lymphocytes in the BAL fluid of patients with sulfur mustard gas-induced pulmonary fibrosis. Respir Med. 2007 Apr 1;101(4):786–92.

4. King AA, Nguyen D, Ionides EL. Statistical inference for partially observed Markov processes via the R package pomp. J Stat Softw. 2016;69(12).

5. Nelder JA, Mead R. A Simplex Method for Function Minimization. Comput J. 1965 Jan 1;7(4):308–13.

6. Leander RN, Wu Y, Ding W, Nelson DE, Sinkala Z. A model of the innate immune response to SARS-CoV-2 in the alveolar epithelium. R Soc Open Sci. 2021 Aug 11;8(8):210090.
